# Impact of COVID‐19 lockdowns on mental health: Evidence from a quasi‐natural experiment in England and Scotland

**DOI:** 10.1002/hec.4453

**Published:** 2021-11-12

**Authors:** Manuel Serrano‐Alarcón, Alexander Kentikelenis, Martin Mckee, David Stuckler

**Affiliations:** ^1^ DONDENA Centre for Research on Social Dynamics and Public Policy Bocconi University Milano Italy; ^2^ Department of Social & Political Sciences Bocconi University Milano Italy; ^3^ Department of Health Services Research and Policy London School of Hygiene and Tropical Medicine University of London London UK

**Keywords:** COVID‐19, health, health inequality, lockdown, mental health

## Abstract

The COVID‐19 pandemic has been associated with worsening mental health but it is unclear whether this is a direct consequence of containment measures, like “Stay at Home” orders, or due to other considerations, such as fear and uncertainty about becoming infected. It is also unclear how responsive mental health is to a changing situation. Exploiting the different policy responses to COVID‐19 in England and Scotland and using a difference‐in‐difference analysis, we show that easing lockdown measures rapidly improves mental health. The results were driven by individuals with lower socioeconomic position, in terms of education or financial situation, who benefited more from the end of the strict lockdown, whereas they suffered a larger decline in mental health where the lockdown was extended. Overall, mental health appears to be more sensitive to the imposition of containment policies than to the evolution of the pandemic itself. As lockdown measures may continue to be necessary in the future, further efforts (both financial and mental health support) are required to minimize the consequences of COVID‐19 containment policies for mental health.

## 1 INTRODUCTION

There is emerging evidence that the COVID‐19 pandemic and accompanying policy responses are generating a mental health crisis in Europe (Arendt et al., 2020; Banks & Xu, 2020). The declines in mental health began during the first months of the pandemic in March–May 2020 (Banks & Xu, 2020; Pierce et al., 2020), and were concentrated among women, youth, people with caring duties, and those reporting financial difficulties (Banks & Xu, 2020; Cheng et al., 2020; Pierce et al., 2020).

Yet the reasons for this deterioration are debated. One potential explanation is that, in a pandemic, mental health will deteriorate due to fear and anxiety about one's health and well‐being (Le & Nguyen, 2021). An alternative hypothesis is that it is not the pandemic per se but policy responses to it – and specifically severe mobility restrictions – that trigger worsening mental health. It is plausible that both have played a role.

Early studies documenting the decline of mental health status have faced challenges in differentiating alternative explanations. This is primarily due to the contemporaneity of the pandemic and associated containment measures, making it difficult to identify an appropriate “control” or comparison group (Arendt et al., 2020; Banks & Xu, 2020; Chandola et al., 2020; Pierce et al., 2020; Silverio‐Murillo et al., 2020). One study documented an association between mobility restriction policies and mental health decline, but it could not establish causality since it lacked pre‐COVID data (Devaraj & Patel, 2020). Another important study by Brodeur et al. (2020) used Google search data, revealing that the timing of lockdown policies across European countries and US states correlated positively with searches for terms related to boredom, loneliness, worry and sadness. Armbruster and Klotzbücher (2020), using data on calls to helplines in four German states, found that calls increased more in states where stricter measures were taken. However, while these studies could take into account pre‐COVID trends, they lacked clinically validated measures of mental health or samples representative of the population. Yet, together, these important studies reveal a clear problem.

In the present article, we take advantage of a quasi‐natural experiment created by variations in timing of policy responses to COVID‐19 in England and Scotland. Both pursued similar containment policies during the early months of the pandemic, but began to diverge on May 13, 2020, when England ended its “Stay at Home” order, while Scotland sustained it until May 29. Importantly for our purposes, this happened despite similar trajectories in the COVID pandemics by the time England eased the restrictions. By using difference‐in‐difference (DiD) methods, we test the hypothesis that the relaxation of mobility restrictions while the threat of infection persists led to recovery of mental health.

Our results show that lifting the Stay at Home order improved the mental health of the population, after a large deterioration observed following the onset of the pandemic. In particular, right after the end of the strict lockdown, mental health bounced back in a magnitude equivalent to 31% with respect to the deterioration observed in the first months of the pandemic. The results were driven by individuals with lower socioeconomic status, in terms of education or financial situation, who benefited more from the end of the strict lockdown, whereas they suffered a larger decline in mental health where the lockdown was extended. This suggests that protracted lockdown policies might exacerbate pre‐existing socioeconomic inequalities in mental health.

The remainder of this article is structured as follows. Section 2 describe the data sources and the empirical strategy. Section 3 describes the results. Section 4 discusses the main findings and Section 5 concludes.

## 2 METHODS

### 2.1 Data

We used the UK Household Longitudinal Study (UKHLS), covering waves 9 (fieldwork: 2017–2019), 10 (2018–2020), Covid survey wave 1 (Late April 2020), Covid survey wave 2 (Late May 2020), Covid survey wave 3 (Late June 2020) and Covid survey wave 4 (Late July 2020). The UKHLS sample was selected in two stages. The first stage involved selecting a random sample of postcode sectors as primary sampling units (PSU), with probability proportional to the number of residential addresses in the sector. Then, addresses were selected using systematic random sampling. All residents at each address at the time the field interviewers made contact were identified as sample members (Lynn, 2009). The Covid questionnaires were implemented as a web survey, whereas wave 9 and 10 were a mix of face‐to‐face and wave survey. Panel participants in the UKHLS were sent a message (by mail, SMS or post) inviting them to complete the Covid monthly questionnaires. 46.7% of those who completed the wave 9 (baseline), responded to the Covid survey wave 1 (Late April 2020) (Institute for Social and Economic Research, 2020). This gives a final balanced sample of 9079 individuals (8164 from England, and 915 from Scotland), followed over the six waves (see Sample Selection Flowchart in Supporting Information S1). In order to control for non‐response over waves in our analysis, and obtain results that are representative of the England and Scotland population (Kaminska & Lynn, 2019), we use inverse probability weights (IPWs) as explained in Subsection 2.3.

We measured the mental health of the population using the General Heath Questionnaire (GHQ‐12). This instrument has been used extensively to measure the mental health of many populations (Kashyap & Singh, 2017; Pierce et al., 2020). GHQ‐12 is formed by 12 items, measuring mental health problems on a 4‐point scale (1‐Not at all, 2‐No more than usual, 3‐Rather more than usual, 4‐Much more than usual)^1^. Following previous literature (Banks & Xu, 2020; Chandola et al., 2020), GHQ score was measured on a Caseness scale (referred to as “GHQ‐caseness” from now on), giving a point to each dimension with a score higher than 3, so that the score varies from 0 (best mental health state) to 12 (worst mental health state).

### 2.2 Quasi‐natural experiment design

**FIGURE 1:**
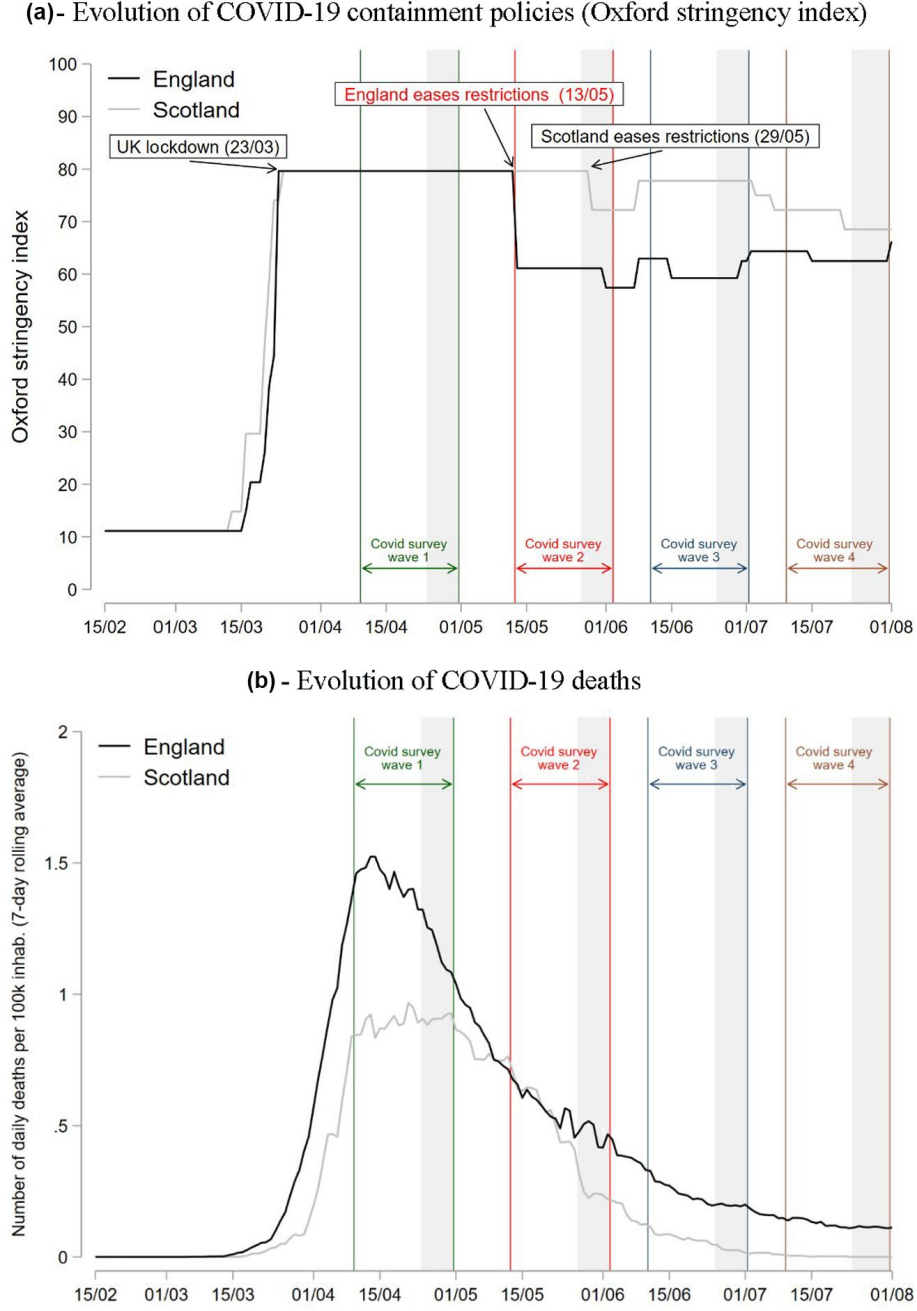
Natural experiment setup. Figure (a) reports the evolution of the Oxford stringency index which measures the strictness of lockdown‐style closure and containment policies that restrict people's behavior (Hale et al., 2020). Shaded areas represent the fieldwork days for each UK Household Longitudinal Study survey wave. The areas between the colorful vertical lines represent the reference period for each survey wave regarding the mental health questions. Mental health questions were framed as how the respondent was feeling “over the last few weeks”. Then, we assumed a reference period of two weeks prior to answering the survey, so that if for wave Covid 2 the fieldwork dates were between May 27 and June 02, then the reference period would be May 13–June 02. Important to note that most of the interviews in Scotland at Wave 2 (68%) were carried out during the days May 17 and 28 when the region was still at the maximum level of restrictions. Figure (b) reports the 7‐day rolling average of COVID‐19 deaths based on the publish date. Data source: UK government (https://coronavirus.data.gov.uk/details/download). (a) Evolution of COVID‐19 containment policies (Oxford stringency index); (b) Evolution of COVID‐19 deaths

In March 23 the UK began a countrywide lockdown under its Stay at Home order, allowing people to only leave home for shopping for basic goods, to obtain medical care, to provide care to others, exercise, or travel to work when working at home was not possible (Hale et al., 2020). This can be seen in a parallel, sharp increase in both nations in the Stringency Index from the Oxford COVID‐19 Government Response Tracker (Figure 1a), which measures the strictness of the lockdown and containment policies (Hale et al., 2020).

Importantly, containment policies started to diverge on May 13, when England ended the Stay at Home order. This ended internal traveling restrictions, allowing individuals to travel anywhere inside England as long as they remained outdoors and did not stay overnight (UK Government, 2020). It also allowed some businesses such as garden centers to reopen (Hale et al., 2020). Furthermore, it allowed people to use parks and socialize in public spaces with at maximum one person outside the household (Quinn, 2020). This included outdoors sport facilities, which were reopened (UK Government, 2020). Overall, this corresponded to 20‐point drop (i.e., 25%) on the Stringency Index. In contrast, Scotland maintained the Stay at Home order until May 29, when outdoor work, non‐contact outdoor leisure activities, and traveling for recreational activities were allowed (Scotish Government, 2020). We take advantage of the timing of the waves of the UKHLS survey relative to the difference in restrictions by UK region. In Covid survey wave 1, both England and Scotland were at the highest level of restriction, whereas by Covid survey wave 2 England had eased the restrictions while Scotland was still at the highest level of restrictions (Figure 1a).

As we can see in Figure 1b, where we report the 7‐day rolling average COVID‐19 deaths per region, by the time England eased restrictions it had about the same number of daily deaths as Scotland. Therefore, we argue that the decision to end the Stay at Home order was not due to England having better COVID‐19 rates than Scotland, but rather reflected different policy approaches to the pandemic. It is possible that the pandemic's evolution could confound our results but, for this to happen, England should have a significantly greater reduction in the death rate between Covid survey waves 1 and 2 (when restrictions were lifted), compared to Scotland. However, the death rate in England has continued to be higher than in Scotland for most of this period.^2^ Still, in the robustness checks in Section 4.2, we control for different measures of pandemic's evolution (both deaths and cases) and our main results hold.

We also test whether people actually complied with the lockdown measures by using Google mobility data and confirm that this was indeed the case (Figure A2 in Supporting Information S1). Individuals in England relatively increased their mobility vis‐à‐vis transit, work or parks, and reduced residential time after England eased restrictions, coinciding with the Covid Survey wave 2. This supports our natural experiment setting.

### 2.3 Statistical analysis

We use a DiD model, including individual fixed effects, as follows:

(1) $${\text{GHQ}}_{i,t}=\beta_{0}+\beta_{1}{\text{Wave}}_{t}+\beta_{2\;}\left({\text{England}}_{i}\times {\text{Wave}}_{t}\right)+X_{it}\gamma +\alpha_{i}+\mu_{i,t}$$

where ${\text{England}}_{i}$ equals 1 if the respondent lived in England by May 2020, and 0 in Scotland^3^. ${\text{Wave}}_{t}$ includes wave fixed effects, leaving Covid Survey wave 1 (Late April) as the base category. $X_{it}$ is a vector of available time‐varying control variables including age measured as continuous variable, and a dummy variable indicating whether the respondent lives alone. $\alpha_{i}$ are individual fixed effects that control for all individual characteristics that are fixed over time. Standard errors are clustered at the individual level. Our coefficient of interest is included in $\beta_{2}$ which reports the interaction between our “treatment” variable (i.e., England) and the wave (i.e., time) dummies. If the earlier easing by England from May 13 had any impact on mental health such interaction should come out significant at Covid survey wave 2, in late May 2020. Additionally, in order to comply with the parallel trends assumption, the interaction coefficient should not be significant in the waves prior to the pandemic. Otherwise, this would indicate that mental health trends had already followed different trajectories across nations prior to the divergence in containment policies.

The outcome variable ${\text{GHQ}}_{i,t}$ uses a Caseness scale (“GHQ‐caseness”) which varies from 0 (best mental health state) to 12 (worst mental health state), as explained in the Data Subsection 2.1. We cluster the standard errors at PSU, which in UKHLS is the postcode sector, as explain in the Section 2.1. Clustering at PSU level allow us to make population inferences that take account of sampling design (Abadie et al., 2017), while at the same time it accounts for correlation of individual errors over time since all individuals belong to the same PSU over the UKHLS waves.

We use IPWs to account for attrition and non‐response bias between waves. These were created by estimating the probability of responding in all waves of the GHQ questionnaire^4^ (i.e., taking part of the balanced sample) as a function of observable variables at the baseline wave (i.e., wave 9): age, age squared, sex, education level, labor market status, self‐reported health status, smoking status, access to Internet, region and household income quintile. IPWs were then formed by the inverse of the predicted probability of responding in the balanced sample. Lastly, we multiplied these weights by the cross‐sectional weights from the wave 9 provided by UKHLS, following the UKHLS indications (Kaminska & Lynn, 2019). As a result, our final balanced sample may be considered representative of those in the England and Scotland population who did not die or move out of the country between wave 9 (2017–2019) and July 2020. More details about the construction of these weights can be found in Supporting Information S1.

## 3 RESULTS

In Table 1 we present Summary statistics at Covid survey wave 1 (i.e., the wave prior to England easing restrictions). Both England and Scotland reported similar levels of mental health (GHQ‐caseness = 2.56 in England vs. 2.68 in Scotland). Our sample is also balanced between the two nations in terms of age and gender. Additionally, both nations show similar loneliness and employment probabilities. There is only a small difference on the percentage of individuals living alone, being that higher in Scotland (16% vs. 12%). Overall, there do not seem to be significant underlying differences between the two nations that could explain our results.

**TABLE 1:** Summary statistics of the balanced sample at Wave Covid 1 (Late April 2020)

	England		Scotland			Total		
	Mean	SD	Mean	SD	*p*‐value^a^	Mean	SD	Missing values
Outcome variable: Mental health								
GHQ‐caseness scale (0–12)	2.56	3.14	2.68	3.19	0.123	2.57	3.14	0
GHQ‐likert scale (0–36)	12.05	5.80	12.34	5.88	0.079	12.08	5.81	0
GHQ‐binary (=1 if any GHQ dimension = 4)	0.22	0.41	0.23	0.42	0.240	0.22	0.41	0
GHQ‐binary 2 (=1 if GHQ‐caseness ≥ 4)	0.27	0.44	0.28	0.45	0.150	0.27	0.44	0
Covariates								
Age	54.88	15.59	55.58	15.05	0.095	54.95	15.54	0
Female (dummy)	0.58	0.49	0.59	0.49	0.387	0.58	0.49	0
Feeling lonely (dummy)	0.33	0.47	0.33	0.47	0.413	0.33	0.47	0
Living alone (dummy)	0.12	0.32	0.16	0.36	0.001	0.12	0.32	0
Employed (dummy)	0.57	0.50	0.58	0.49	0.335	0.57	0.49	14
*n*	8164		915			9079		

*Note*: Balanced sample is formed by individuals with information on mental health variables at waves 9 (2017–2019), 10 (2018–2020), Covid 1 (Late April 2020), Covid 2 (Late May 2020), Covid 3 (Late June 2020) and Covid 4 (Late July 2020).

Abbreviation: GHQ, General Heath Questionnaire.

^a^ *p*‐value from a *t*‐test of the difference in means between England and Scotland.

**FIGURE 2:**
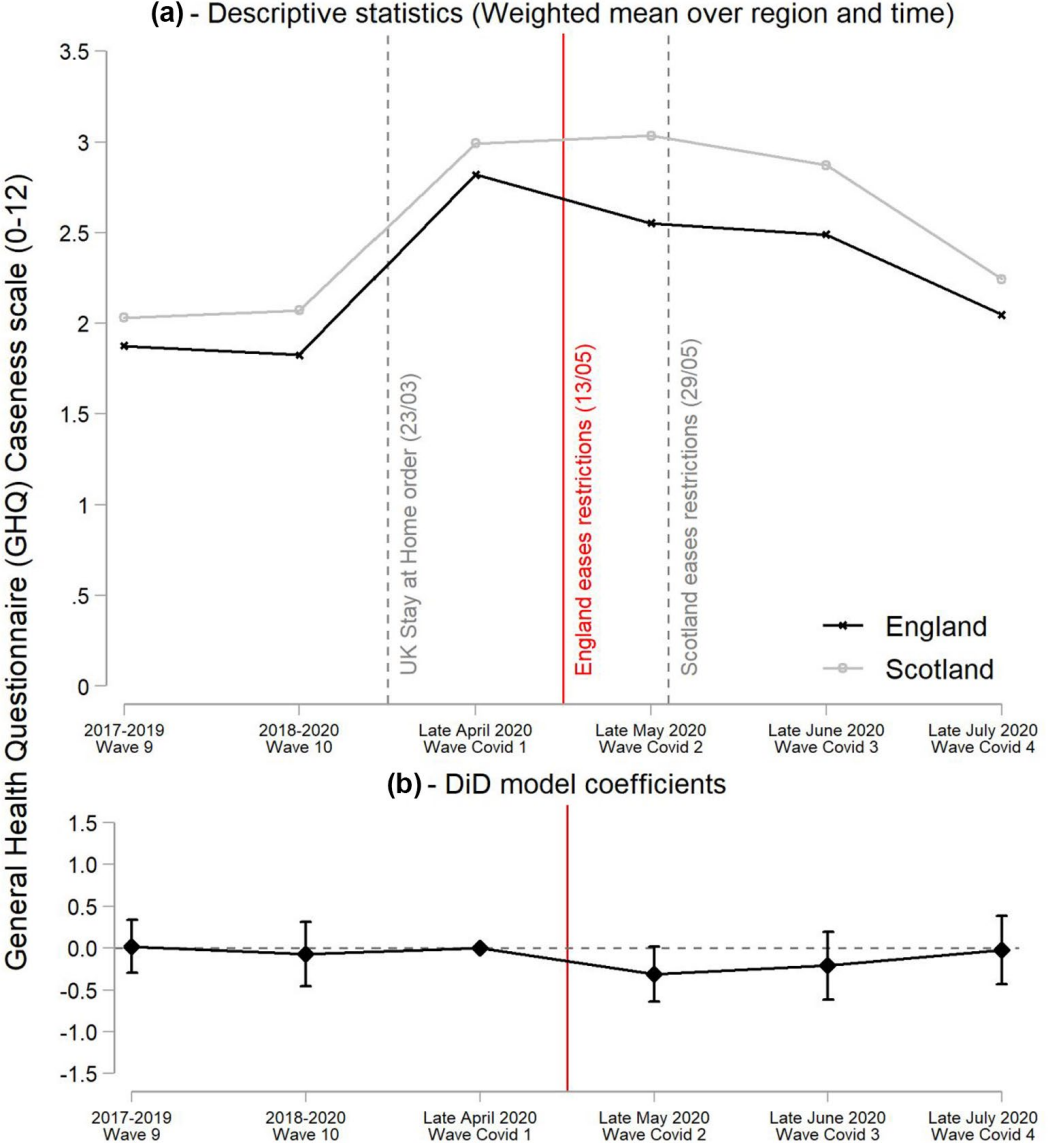
Difference‐in‐difference (DiD) results of the effect of easing restrictions on General Health Questionnaire (GHQ)‐caseness. Figure A reports the weighted mean of the GHQ‐caseness scale over nation and UK Household Longitudinal Study wave. Figure B reports the coefficients and the 95% confidence intervals of the interaction between the England dummy and the wave dummies ($\beta_{2\;}$ in model of Equation 1), leaving survey wave Covid 1 (Late April) as base category. Full results of this model are reported in Table A1 of Supporting Information S1 (Column [3]). (a) Descriptive statistics (Weighted mean over region and time); (b) DiD model coefficients

As shown in Figure 2a, mental health deteriorated by a similar magnitude in both nations following the onset of the pandemic and the UK wide Stay at Home order. However, by late May, at Covid survey wave 2, mental health started to bounce back in England following the end of the Stay at Home order. On the other hand, it continued to deteriorate in Scotland, not improving until June, after its restrictions began to be eased. From then on, it starts to catch up with England. By late July, mental health was continuing to improve in both nations until almost reaching pre‐pandemic levels.

The DiD results show that easing restrictions in England by mid‐May is associated with improved mental health, with a reduction by 0.31 points, albeit at a lower threshold of statistical significance (*p* < 0.10) in the GHQ‐caseness score (Figure 2b). This amounts to a 31% reduction with respect to the first increase in GHQ‐caseness observed by April 2020, following the onset of the pandemic. Full results of the model are reported in Table A1 of the Supporting Information S1. Our preferred model is that shown in Column (3) which includes age and living alone as control variables. Interactions between the England dummy and the dummies of the waves prior to the pandemic (Waves 9 and 10) are not significant and coefficients are very close to zero, confirming that the trends in mental health were parallel in England and Scotland prior to England easing restrictions.

### 3.1 Results by GHQ dimension

**FIGURE 3:**
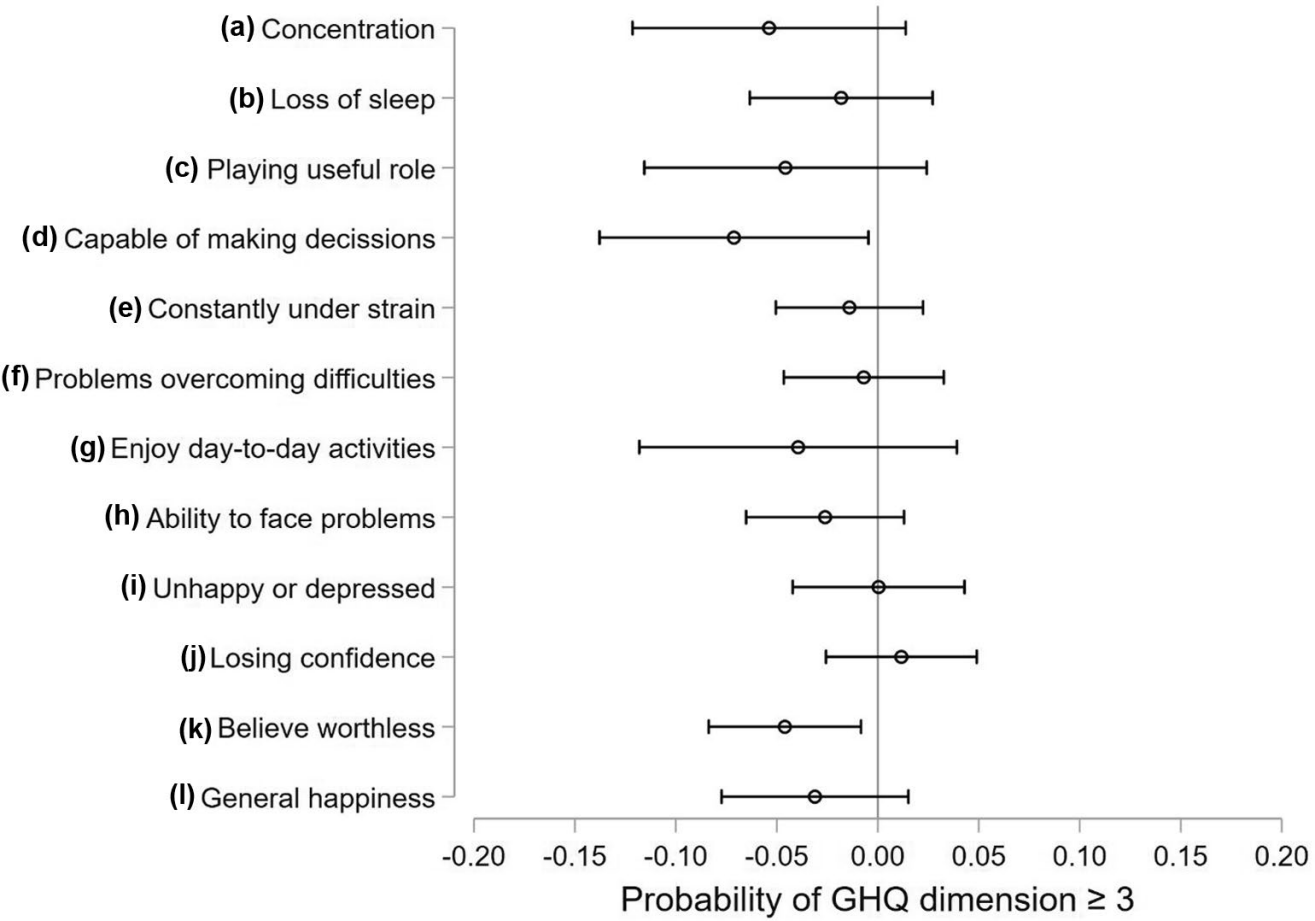
Difference‐in‐difference (DiD) effect of easing lockdown by dimension of General Health Questionnaire (GHQ)‐caseness. This figure reports the coefficients and the 95% confidence intervals of the interaction between the England dummy and the Covid survey wave 2 dummy (within $\beta_{2}$ in model of Equation 1). Each coefficient comes from a different regression where the dependent variable is equal 1 if the correspondent GHQ dimension is equal to 3 or 4, and zero otherwise. Full results of these regressions are reported in Table A2 of Supporting Information S1

In Figure 3, we report the DiD results by the 12 dimensions of GHQ. In particular, the improvement in mental health associated with lifting the Stay at Home order in England seems to be driven by an improvement in the “capable of making decisions” and “believing yourself worthless” components. However, we believe that it is not appropriate to place too much weight on the results associated with individual components as most of the coefficients are not statistically significantly different, with confidence intervals overlapping. Instead, we suggest that the more important finding is that all but two dimensions seem to be negatively affected by the lockdown measures, which would point to a general improvement across many dimensions of mental health associated with their easing.

### 3.2 Results by socioeconomic group

**FIGURE 4:**
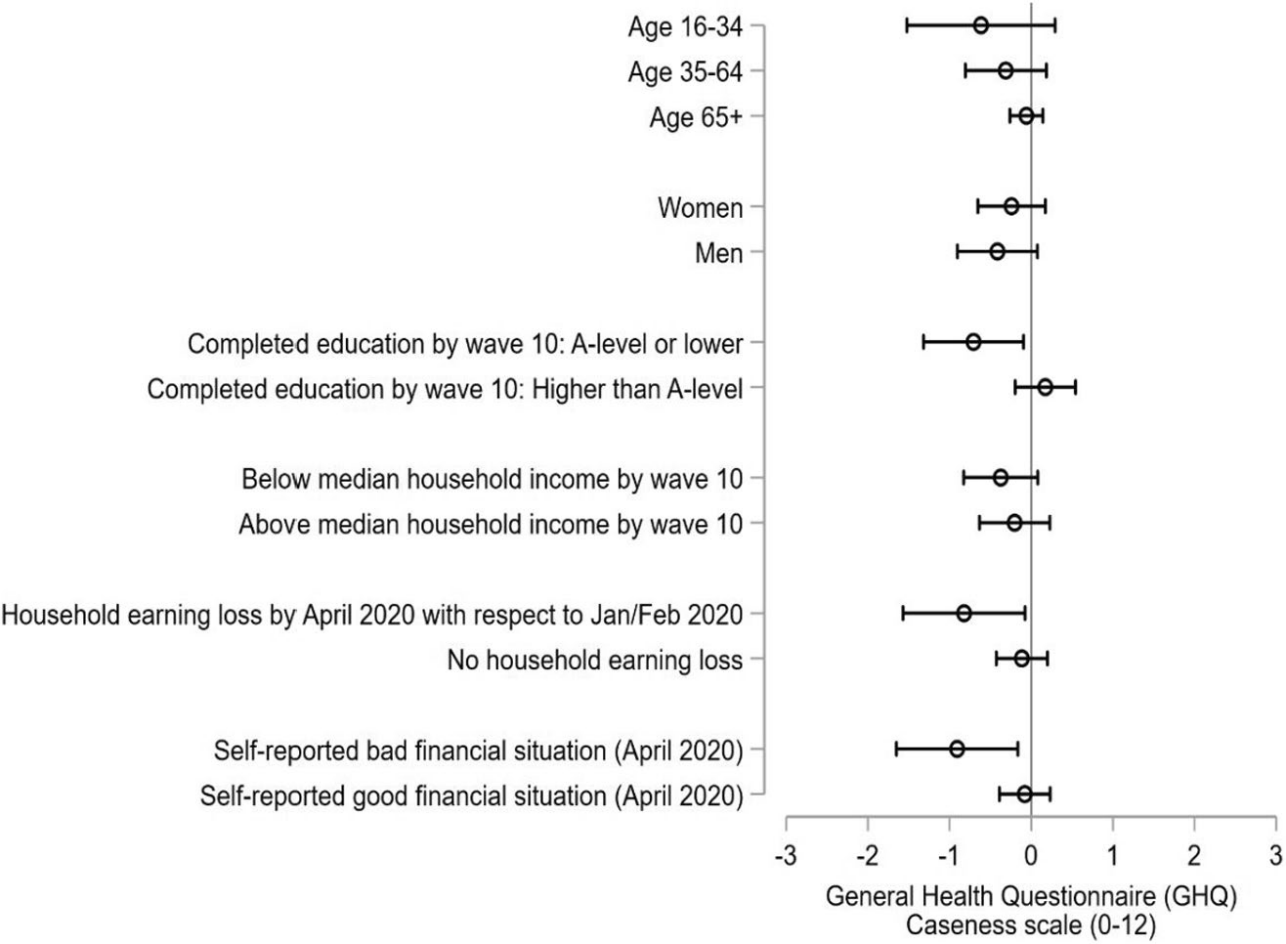
Difference‐in‐difference (DiD) effect of easing lockdown by socioeconomic group. General Health Questionnaire (GHQ)‐caseness. This figure reports the coefficients and the 95% confidence intervals of the interaction between the England dummy and the Covid survey wave 2 dummy (within $\beta_{2}$ in model of Equation 1). Each coefficient comes from a different regression using the corresponding subsample of each socioeconomic group. Age groups are based on respondent age by April 2020. Full results of these regressions are reported in Tables A3 and A4 of Supporting Information S1. Household earning loss is based on the question “Is your household is now earning less than in January/February 2020?”. Self‐reported financial situation is based on the question “How well would you say you yourself are managing financially these days?”; those who responded “living comfortably” or “doing alright” were classified as Good financial situation, whereas those who responded “Just about getting by”, “Finding it quite difficult” or “Finding it very difficult” were classified as Bad financial situation. The evolution of the mean GHQ‐caseness by socioeconomic group and nation is reported in Figures A3–A8 of Supporting Information S1

In Figure 4 we report the effects of easing lockdown on mental health, by age, sex and socioeconomic group, derived from a subsample analysis of the DiD model. First, results do not seem to differ by age group^5^ and sex. On the other hand, the easing of restrictions relatively improved the mental health of those with lower education, but not of those with higher education. Respondents who were economically affected by the pandemic in the first months seem to be the most relieved by the easing of restrictions. The mental health of those suffering an earning loss in April and those who report a bad financial situation largely improved as a consequence of the end of the Stay at Home order in England, whereas the effect was not significant for those who were not suffering financial difficulties. We further test whether this differential effect of easing restrictions by socioeconomic group was statistically significant using a triple difference model (See Supporting Information S1). The triple interaction (i.e., socioeconomic group × England × Covid Wave 2) was significant for education, suggesting that the effect of easing restrictions was significantly greater for those with lower education. Similarly, the triple difference coefficient was significant for those reporting a bad financial situation, whereas it did not reach significance for different degrees of household earnings loss (Table E1 in Supporting Information S1).

Looking simply at the evolution of the mean GHQ‐caseness score by region and socioeconomic group (Figures A3–A6 in Supporting Information S1) is insightful. By April 2020, following the onset of the pandemic and UK‐wide Stay at Home order, both those with a low and high socioeconomic status as measured by education, income or individual financial situation experienced declines in their mental health. However, after England eased restrictions, the mental health of people in England who were disadvantaged in these respects improved, whereas it continued to deteriorate for their equivalents living in Scotland, who still were under the Stay at Home order. Among the more advantaged group, however, those in both Scotland and England had experienced improvements in their mental health by May 2020. Overall, this shows that easing the Stay at Home order particularly benefited those who were already disadvantaged and did so rapidly, whereas those who were advantaged started to bounce back in the longer term independently of the level of restrictions.

### 3.3 Exploring the potential causal channels

So far we have not explored the channels through which the easing of restrictions may have improved the mental health of the population. One of the reasons why lockdown may affect mental health is through social isolation (Loades et al., 2020). We intend to test this channel by using the same DiD model as in Equation (1), but now with the probability of feeling lonely as the dependent variable. If the easing of restrictions led to a reduction in loneliness, our DiD coefficient of interest (England × Covid wave 2) should be negative and significant. However, results from Table 2 Column 1 suggest that there was no effect of easing restrictions on the probability of feeling lonely, with the coefficient being even positive. Still, we should not interpret this as a proof that relaxing lockdown measures did not alter social isolation since we lack other more direct measures of social isolation such as the number, frequency or closeness of social connections (Cornwell & Waite, 2009).

**TABLE 2:** DiD models to test the potential causal channels

Variables	(3)	(3)	(3)
	Pr (Feel lonely)	Pr (Employed)	Pr (Working zero hours)
DiD interactions			
(Base category: Wave Covid 1 – April 2020)			
England × Wave 9 (2017–2019)	0.0787	0.0214	‐
	(0.057)	(0.018)	
England × Wave 10 (2018–2020)	0.0470	0.0318*	‐
	(0.037)	(0.018)	
England × (Janurary/February 2020)	‐	‐	0.0243
			(0.047)
**England** **×** **Wave Covid 2 (May 2020)**	0.0211	−0.00210	−0.0319*
	(0.029)	(0.005)	(0.018)
England × Wave Covid 3 (June 2020)	0.0430	0.000480	−0.00330
	(0.039)	(0.007)	(0.050)
England × Wave Covid 4 (July 2020)	0.0620	0.00593	−0.00139
	(0.038)	(0.011)	(0.055)
Age	−0.0149	0.00436	−0.0155
	(0.010)	(0.009)	(0.012)
Living alone	−0.0177	−0.00397	−0.0309
	(0.021)	(0.010)	(0.019)
Wave fixed effects	Yes	Yes	Yes
Individual fixed effects	Yes	Yes	Yes
Observations	47,934	47,897	22,068
Number of individuals	7991	7991	4589

*Note*: Each column reports results from a different regression. Robust standard errors clustered at primary sampling unit in parentheses ***p < 0.01, **p < 0.05, *p < 0.1. In column (1) the dependent variable is the probability of feeling lonely “often” or “some of the time”. In column (2) the dependent variable is the probability of being employed. In column (3) we use the subsample of those who declare to have been employed right before the pandemic, in January/February 2020. We get that information retrospectively from survey wave Covid 1 (April 2020). The dependent variable is the probability of declaring to be working zero hours. Note that from 1544 employed respondents who are in our sample declared to be working zero hours by April 2020, 838 (54%) were in furlough, 253 (16%) declared to have their business affected by the Covid‐19 containment regulations. Information about the reasons why they are working zero hours is only available in Wave Covid 1 (April 2020). Note that DiD time variables are slightly different in the Column (3) model. Information on working zero hours was not available in Wave 9 and Wave 10. However, we do have information on working zero hours in January/February 2020. As a result the DiD model for working zero hours has 4 time dummies: January/February 2020, Wave Covid 2 (May 2020), Wave Covid 3 (June 2020) and Wave Covid 4 (July), leaving again Wave Covid 1 (April 2020) as the baseline category. The interaction in bold measures the effect of easing lockdown restrictions on each corresponding dependent variable.

Abbreviation: DiD, difference‐in‐difference.

On the other hand, results from the previous section point towards the importance of economic factors in explaining our results since those already disadvantaged and suffering financial difficulties were the most affected by the lockdown extension. To further explore this channel, we carried out the same DiD model but using as a dependent variable the probability of employment. The early easing of restrictions might have increased economic activity and job opportunities, which may end up improving mental health, especially on those suffering from financial difficulties. If that is the case, our DiD coefficient of interest (England × Covid wave 2) should be positive and significant for the probability of employment. However, results from Table 2 Column 2 suggest that there was no effect of relaxing restriction on the probability of employment^6^. Actually, as we can see in Figure A9 in Supporting Information S1, the probability of employment barely changed during the period of analysis.

However, even if individuals did not experience job loss, they may still have been affected by the pandemic. For instance, they may have been furloughed (with a corresponding pay cut and spending more time isolated at home) or with reduced activity of their business (if self‐employed). In order to create a proxy to capture this effect we use now the probability of declaring working zero hours as dependent variable in a similar DiD setting^7^. Note that from 1544 employed respondents who reported working zero hours by April 2020, most were due to economic effects of the pandemic: 838 (54%) were on furlough and 253 (16%) reported having their business affected by the COVID‐19 containment regulations^8^. As reported in Table 2 Column 3, the probability of working zero hours seem to decrease by around 3 percentage points (*p* < 0.10) with the easing of restrictions. This suggests that even if the probability of employment remained unchanged with the different lockdown policy responses, economic activity was still altered by the different levels of restrictions, which may have end up affecting mental health.

## 4 ROBUSTNESS CHECKS

### 4.1 Alternative measures of mental health

We carried out several robustness checks to our analysis. First, we employed alternative measures of mental health following earlier studies (Banks & Xu, 2020; Davillas & Jones, 2021; Jackson, 2007; Thomson et al., 2018): (i) a Likert scale from 0 to 36 (“GHQ‐likert”), (ii) a binary indicator of whether any item has the maximum score of 4 (“GHQ‐binary”), and (iii) a binary indicator of whether the individual reports the two most symptomatic answers (out of the 4 possible) in at least 4 of the 12 GHQ dimensions (“GHQ‐binary2”).^9^ Our main indicator, GHQ‐caseness, and GHQ‐likert may be thought of measures of the level of mental well‐being of the population, whereas both GHQ‐binary and GHQ‐binary2 are more extreme measures of mental health and can be thought of a proxy for suffering mental health disorders (Jackson, 2007; Thomson et al., 2018). GHQ‐likert presents exactly the same results than GHQ‐caseness (Table A6 in Supporting Information S1), suggesting that there was an improvement in the mental well‐being of the population following the easing of restrictions and that this was particularly driven by individuals with lower levels of education and experiencing financial difficulties due to the pandemic (Tables A7 and A8 in Supporting Information S1). Results using the more extreme measures of mental health are somewhat contradictory. Whereas the effect on GHQ‐binary is strongly significant (*p* < 0.01) both in the general model (Table A9 in Supporting Information S1), and for those in a more disadvantaged position (Tables A10 and A11 in Supporting Information S1) results turn insignificant when using GHQ‐binary2, even though the coefficient of the interaction of interest remains negative (Table A12 in Supporting Information S1). This may reflect disagreement about which threshold to use when using the GHQ to create a binary indicator for mental health disorders (Maheswaran et al., 2015). Still, the main objective of this article is to test the causal effect of lockdown on the overall mental health level of the population, rather than on the prevalence of mental health disorders.

### 4.2 Control by the evolution of the pandemic

Second, even though the trajectory of the pandemic was similar in each nation when England opened up (although by early summer rates in England were somewhat higher in England) (Figure 1b), we still carry out some robustness checks to confirm that a differential evolution of the pandemic is not driving our results. Specifically, we alternatively add several variables to our main model: (i) 7‐day rolling average of daily cases by publish date, (ii) 7‐day rolling average of daily cases by publish date by specimen date, (iii) cumulative number of cases, (iv) 7‐day rolling average of death, and (v) cumulative number of deaths. We use both cases and deaths since the latter might be a closer measure of the evolution of the pandemic in the first months considering the low testing capacities at the beginning of the pandemic. More details about these variables and how they were inserted in the model can be found in Supporting Information S1. As shown in Tables A13–A16 in Supporting Information S1, our results do not change with the inclusion of these variables at the four mental health outcome variables (i.e., GHQ‐caseness, GHQ‐likert, GHQ‐binary, GHQ‐binary2). Additionally, most of the coefficients of the variables adjusting for the evolution of the pandemic are not statistically significant. Overall, this suggests that mental health is more sensitive to containment policies, rather than to the evolution of the pandemic.

### 4.3 Fieldwork days of Covid survey wave 2 (Late May 2020)

As discussed in the section on the natural experiment design, Scotland eased some national restrictions 29 May, by allowing outdoor work and non‐contact outdoor leisure activities, and therefore de facto ending the Stay at Home order. As shown in Figure 1, this was in between the fieldwork days of the Covid survey wave 2. Fieldwork days during that wave were May 27–June 02, and 32% of the respondents in Scotland actually carried out the survey after the restrictions were lifted (i.e., May 29–June 02). Still, this is unlikely to affect our identification strategy since the reference period of our variable of interest (i.e., GHQ scores) is at least two weeks before the date that responses were recorded (the questions were framed as how the respondent was feeling “over the last few weeks”). Then, during most of the reference period in Covid survey wave 2, the Stay at Home order was still in place in Scotland, but not in England. Still, we carry out a robustness check by dropping from our estimations the 32% of respondents in Scotland who responded to Covid survey wave 2 after the restrictions were lifted in Scotland (i.e., 293 out of 915 Scotland respondents of our final sample). Coefficients hardly changed despite the drop in the sample size (Table A17 in Supporting Information S1).

## 5 DISCUSSION

In this article, we sought to ascertain the causal effect of COVID‐19 containment policies on population mental health in a quasi‐natural experiment setting by comparing the policy responses of England and Scotland. Our results show that lifting the Stay at Home order improved mental health among the population (particularly among the economically worse‐off), after a large deterioration observed following the onset of the pandemic. Our results therefore suggest that mental health may bounce back once strict lockdown measures are lifted, and show mental health to be more sensitive to the imposition of containment policies than to the evolution of the pandemic itself (Le & Nguyen, 2021), at least within the range observed in these two nations. We cannot, however, assume that this would still be the case if cases and deaths had risen to very high levels after restrictions were removed.

The results are particularly important considering that when England ended the Stay at Home order, many restrictions remained in place. Compared to Scotland, socializing in public places was allowed, but only with one person from outside the household. Also, some businesses were encouraged to reopen but bars, retail stores, theaters, gyms and all leisure facilities remained closed. Lastly, although internal traveling restrictions were removed, it was still not allowed to stay in elsewhere for a holiday (UK Government, 2020). Therefore, our results indicate that lifting the strict lockdown while maintaining relatively strong containment measures can already significantly ease the mental health burden of the population in a short period of time.

The deterioration of mental health following the first months of the pandemic has been reported to be worse among younger people and women (Banks & Xu, 2020; Pierce et al., 2020). However, our results show that easing the lockdown did not have a differential effect by age or gender. Previous studies were inconclusive as regards pre‐existing levels of disadvantage. Some studies (Chandola et al., 2020; Etheridge & Spantig, 2020) showed that the deterioration in mental health was greater among those in a worse financial situation, whereas Davillas and Jones (2021) noted that the pandemic initially acted as a leveler in that it was associated with a similar deterioration in mental health for all groups. Our results show that easing the lockdown restrictions benefited more those already disadvantaged in terms of education and financial difficulties. Or to put another way, those already disadvantaged suffered a larger deterioration in mental health with the prolongation of the Stay at Home order. This suggests that persistent lockdown policies might exacerbate pre‐existing socioeconomic inequalities in mental health.

Our analysis also has some limitations. First, while we use a widely used and validated measure of population mental health, it is not clear whether the same pattern would be seen with other measures of mental health outcomes, such as medicine consumption or healthcare use. In fact, healthcare contacts for mental health conditions have been shown to decrease following the onset of the pandemic (Mansfield et al., 2021). Future research might cast light on whether the difference in policy responses by nation also had an impact on the use of mental healthcare services and whether this translates or not into differential unmet mental health needs. Second, even though our results point towards the importance of economic factors mediating the effect of easing lockdown on improved mental health, we lacked more comprehensive indicators of social connections and social isolation, which could be used in the future to ascertain whether a reduction in social isolation has also played an important role. Another possibility is that our results could be driven by a misperception of residents in England that restrictions were lifted because they had lower cases/deaths than Scotland. However, we find this unlikely considering that news of the progress of the pandemic was covered widely in the media. Therefore, we expect that individuals were relatively well‐informed on the real evolution of the pandemic.

## 6 CONCLUSION

Restrictions on movement were essential to reduce transmission of the COVID‐19 virus and may also become necessary in the future given the emergence of new variants of the COVID‐19 virus—some potentially able to escape vaccine‐induced immunity—and uncertainty surrounding vaccination rollout. Our results show that lifting the Stay at Home order was associated with improved mental health, after a large deterioration following the onset of the pandemic. These results were driven by individuals with lower levels of education and experiencing financial difficulties who were particularly affected when the lockdown was extended. Lastly, our results show mental health to be more sensitive to the imposition of containment policies than to the evolution of the pandemic itself. These findings should not be interpreted as supporting the removal of restrictions if it is unsafe to do so. However, they do stress the importance of taking compensatory mechanisms to protect those who are the most likely to suffer from these necessary restrictions.

## CONFLICT OF INTEREST

The authors declare no conflict of interest.

## Supporting information

Supporting Information S1

## Data Availability

The data that supports the findings of this study are openly available in University of Essex, Institute for Social and Economic Research (2021). Understanding Society: Waves 1–10, 2009–2019 and Harmonised BHPS: Waves 1–18, 1991–2009 [data collection]. 13th Edition. UK Data Service. SN: 6614, http://doi.org/10.5255/UKDA‐SN‐6614‐14.

## ENDNOTES

^1^
The 12 items of the GHQ‐12 are: a‐concentration, b‐loss of sleep, c‐playing useful role, c‐capable of making decisions, e‐constantly under strain, f‐problems overcoming difficulties, g‐enjoy day‐to‐day activities, h‐ability to face problems, i‐feeling unhappy or depressed, j‐losing confidence, k‐believe worthless, l‐general happiness. The scale of items that are framed in a positive way (i.e., items a, c, d, g, h, and l) is rephrased to: 1‐More so than usual, 2Same as usual, 3‐Less so than usual, 4‐Much less capable. For the exact wording of each item question you may see the survey questionnaire (University of Essex, 2020. Institute for Social and Economic Research, 2020).

^2^
In Figure A1 of Supporting Information S1 we also report the evolution of the 7‐day rolling average of COVID‐19 cases by publish date. This suggests that England had slightly lower incidence by the time it eased restrictions, although the trends there and in Scotland proceeded largely in parallel until the summer. While not conclusive, this does not support the possibility that there was some differential trend in the evolution of the pandemic by region that could affect mental health at the time that English restrictions were eased. However, it is important to note the limitations of case data at the beginning of the pandemic because of limited testing capacity, although this affected both nations to a similar extent.

^3^
Note that it is not necessary to include in the model the nation dummy (${\text{England}}_{i}$) alone since it is fixed over time and therefore will be absorbed by the individual fixed effects ($\alpha_{i}$).

^4^
In UKHLS the GHQ questionnaire was only completed by those responding the self‐completion adult interview, and not by those whose information was provided by a proxy respondent.

^5^
We grouped individuals into three age groups: 16–34, 35–64 and 65 or older. Scotland sample size (*n* = 915) prevented us from doing smaller age groups.

^6^
In Table A5 of the Supporting Information S1 we used a different approach to do a mediating analysis by adding in a stepwise manner the potential mediating factors (i.e., employment and loneliness) to our main regression. Our DiD coefficients remained stable, both in size and significance, when including these variables, suggesting again that these factors do not mediate the relationship between lockdown policies and mental health.

^7^
Note that DiD time variables are slightly different now. Information on working zero hours was not available in Wave 9 and Wave 10. However, we do have information on working zero hours in January/February 2020. As a result the DiD model for working zero hours has four time dummies: January/February 2020, Wave Covid 2 (May 2020), Wave Covid 3 (June 2020) and Wave Covid 4 (July), leaving again Wave Covid 1 (April 2020) as the baseline category.

^8^
Information about the reasons why they are working zero hours is only available in Wave Covid 1 (April 2020). Therefore, we could not do an analysis disaggregated by reason.

^9^
Note that this binary indicator (“GHQ‐binary2”) is similar to setting a dummy equal one if GHQ‐caseness ≥ 4.
