# Supplementary material for: Impact of COVID‐19 lockdowns on mental health: Evidence from a quasi‐natural experiment in England and Scotland
Source: Health Econ. 2021 Nov 12;31(2):284–96. doi: 10.1002/hec.4453 (PMC8646947; doi:10.1002/hec.4453)
Supplement: Supplementary file 1 — Supporting Information S1 [file HEC-31-284-s001.docx]

**APPENDIX**

**Appendix A. Supplementary results**

Table A1- Difference-in-difference (DiD) results. GHQ-caseness

|  |  |  |  |
| --- | --- | --- | --- |
| VARIABLES | (1) | (2) | (3) |
|  |  |  |  |
| DiD interactions |  |  |  |
| (base category: Wave Covid 1) |  |  |  |
| England x Wave 9 | 0.0183 | 0.0230 | 0.0187 |
|  | (0.161) | (0.162) | (0.162) |
| England x Wave 10 | -0.0735 | -0.0679 | -0.0697 |
|  | (0.197) | (0.197) | (0.197) |
| **England x Wave Covid 2** | -0.309* | -0.310* | -0.311* |
|  | (0.169) | (0.169) | (0.169) |
| England x Wave Covid 3 | -0.209 | -0.210 | -0.211 |
|  | (0.206) | (0.206) | (0.206) |
| England x Wave Covid 4 | -0.0232 | -0.0228 | -0.0237 |
|  | (0.209) | (0.208) | (0.208) |
| Age |  | -0.0343 | -0.0342 |
|  |  | (0.065) | (0.065) |
| Living alone |  |  | -0.0812 |
|  |  |  | (0.136) |
|  |  |  |  |
| Wave Fixed Effects | Yes | Yes | Yes |
| Individual Fixed Effects | Yes | Yes | Yes |
|  |  |  |  |
| Observations | 47,946 | 47,945 | 47,945 |
| Number of individuals | 7,991 | 7,991 | 7,991 |

NOTES: Each column reports results from a different regression, where the dependent variable is the GHQ-caseness (0-12). Robust standard errors clustered at primary sampling unit in parenthesis. *** p<0.01, ** p<0.05, * p<0.1. Note that the difference in observations between the final balanced sample (n=9,079) and the sample used in the regressions (n=7,991) is due to some survey respondents (1,088) having zero weights by sampling design (Kaminska & Lynn, 2019).

Table A2- Difference-in-difference (DiD) results by dimension. GHQ-caseness

|  | | | (1) | | (2) | | (3) | | (4) | | (5) | | (6) | | (7) | | (8) | | (9) | | (10) | | (11) | | (12) |  |
| --- | --- | --- | --- | --- | --- | --- | --- | --- | --- | --- | --- | --- | --- | --- | --- | --- | --- | --- | --- | --- | --- | --- | --- | --- | --- | --- |
| VARIABLES | | | a | | b | | c | | d | | e | | f | | g | | h | | i | | j | | k | | l |  |
|  |  |  | |  | |  | |  | |  | |  | |  | |  | |  | |  | |  | |  |  |  |
| DiD interactions | | |  | |  | |  | |  | |  | |  | |  | |  | |  | |  | |  | |  |  |
| (base category: Wave Covid 1) | | |  | |  | |  | |  | |  | |  | |  | |  | |  | |  | |  | |  |  |
| England x Wave 9 | | | 0.008 | | -0.012 | | -0.040 | | 0.012 | | 0.017 | | -0.032 | | 0.018 | | -0.018 | | 0.061 | | 0.011 | | -0.001 | | -0.005 |  |
|  | | | (0.027) | | (0.023) | | (0.060) | | (0.023) | | (0.037) | | (0.022) | | (0.034) | | (0.025) | | (0.049) | | (0.023) | | (0.018) | | (0.026) |  |
| England x Wave 10 | | | -0.045 | | -0.007 | | -0.006 | | -0.049 | | 0.004 | | -0.043** | | 0.041 | | -0.023 | | 0.065 | | 0.018 | | -0.020 | | -0.005 |  |
|  | | | (0.058) | | (0.022) | | (0.026) | | (0.052) | | (0.038) | | (0.021) | | (0.038) | | (0.022) | | (0.048) | | (0.025) | | (0.018) | | (0.028) |  |
| **England x Wave Covid 2** | | | -0.052 | | -0.016 | | -0.043 | | -0.070** | | -0.012 | | -0.004 | | -0.038 | | -0.024 | | 0.005 | | 0.015 | | -0.044** | | -0.028 |  |
|  | | | (0.035) | | (0.024) | | (0.036) | | (0.034) | | (0.019) | | (0.021) | | (0.041) | | (0.021) | | (0.020) | | (0.017) | | (0.019) | | (0.024) |  |
| England x Wave Covid 3 | | | -0.005 | | -0.027 | | -0.017 | | -0.078 | | 0.003 | | 0.005 | | -0.006 | | -0.027 | | 0.011 | | 0.018 | | -0.024 | | -0.064 |  |
|  | | | (0.025) | | (0.022) | | (0.023) | | (0.053) | | (0.024) | | (0.026) | | (0.029) | | (0.025) | | (0.021) | | (0.021) | | (0.019) | | (0.053) |  |
| England x Wave Covid 4 | | | -0.029 | | -0.010 | | -0.021 | | -0.009 | | 0.028 | | 0.006 | | 0.010 | | -0.019 | | 0.042* | | 0.029 | | -0.002 | | -0.049 |  |
|  | | | (0.057) | | (0.025) | | (0.022) | | (0.020) | | (0.036) | | (0.021) | | (0.030) | | (0.023) | | (0.024) | | (0.018) | | (0.018) | | (0.056) |  |
| Age | | | -0.001 | | 0.002 | | -0.009 | | -0.006 | | 0.001 | | -0.001 | | -0.016* | | -0.004 | | 0.002 | | -0.005 | | 0.005 | | -0.002 |  |
|  | | | (0.011) | | (0.007) | | (0.009) | | (0.008) | | (0.008) | | (0.007) | | (0.009) | | (0.006) | | (0.009) | | (0.007) | | (0.007) | | (0.009) |  |
| Living alone | | | -0.032* | | -0.001 | | -0.009 | | 0.001 | | -0.023 | | -0.012 | | -0.002 | | 0.001 | | -0.003 | | 0.020 | | -0.016 | | -0.003 |  |
|  | | | (0.017) | | (0.018) | | (0.017) | | (0.013) | | (0.019) | | (0.020) | | (0.018) | | (0.015) | | (0.018) | | (0.017) | | (0.015) | | (0.022) |  |
|  | | |  | |  | |  | |  | |  | |  | |  | |  | |  | |  | |  | |  |  |
| Wave Fixed Effects | | | Yes | | Yes | | Yes | | Yes | | Yes | | Yes | | Yes | | Yes | | Yes | | Yes | | Yes | | Yes |  |
| Individual Fixed Effects | | | Yes | | Yes | | Yes | | Yes | | Yes | | Yes | | Yes | | Yes | | Yes | | Yes | | Yes | | Yes |  |
|  | | |  | |  | |  | |  | |  | |  | |  | |  | |  | |  | |  | |  |  |
| Observations | | | 47,945 | | 47,945 | | 47,945 | | 47,945 | | 47,945 | | 47,945 | | 47,945 | | 47,945 | | 47,945 | | 47,945 | | 47,945 | | 47,945 |  |
| Number of individuals | | | 7,991 | | 7,991 | | 7,991 | | 7,991 | | 7,991 | | 7,991 | | 7,991 | | 7,991 | | 7,991 | | 7,991 | | 7,991 | | 7,991 |  |
| NOTES. Each column reports results from a different regression, where the dependent variable is equal 1 if the correspondent GHQ dimension is equal 3 or 4. The 12 dimensions of the GHQ-12 are: a- concentration, b- loss of sleep, c- playing useful role, d-capable of making decisions, e- constantly under strain, f- problems overcoming difficulties, g- enjoy day-to-day activities, h- ability to face problems, i- feeling unhappy or depressed, j- losing confidence, k- believe worthless, l- general happiness. Robust standard errors clustered at primary sampling unit in parentheses *** p<0.01, ** p<0.05, * p<0.1. Note that the difference in observations between the final balanced sample (n=9,079) and the sample used in the regressions (n=7,991) is due to some survey respondents (1,088) having zero weights by sampling design (Kaminska & Lynn, 2019). | | | | | | | | | | | | | | | | | | | | | | | | | | |

Table A3- Difference-in-difference (DiD) results by socioeconomic group (I).

|  | Age ^†^ | | | Sex | |
| --- | --- | --- | --- | --- | --- |
| VARIABLES | 16-34 | 35-64 | 65+ | Women | Men |
|  |  |  |  |  |  |
| DiD interactions |  |  |  |  |  |
| (base category: Wave Covid 1) |  |  |  |  |  |
| England x Wave 9 | 0.648 | -0.140 | -0.077 | -0.070 | 0.152 |
|  | (0.475) | (0.205) | (0.216) | (0.254) | (0.207) |
| England x Wave 10 | 0.253 | 0.028 | -0.420 | 0.094 | -0.203 |
|  | (0.573) | (0.234) | (0.317) | (0.263) | (0.293) |
| **England x Wave Covid 2** | -0.596 | -0.304 | -0.014 | -0.190 | -0.410 |
|  | (0.418) | (0.261) | (0.100) | (0.202) | (0.255) |
| England x Wave Covid 3 | -0.789 | 0.096 | -0.258 | -0.280 | -0.108 |
|  | (0.550) | (0.246) | (0.374) | (0.267) | (0.329) |
| England x Wave Covid 4 | 0.069 | 0.271 | -0.540 | 0.050 | -0.062 |
|  | (0.488) | (0.215) | (0.389) | (0.252) | (0.306) |
| Age | -0.048 | -0.009 | -0.049 | -0.019 | -0.053 |
|  | (0.165) | (0.087) | (0.068) | (0.096) | (0.081) |
| Living alone | 0.115 | -0.310* | 0.130 | 0.043 | -0.241 |
|  | (0.340) | (0.171) | (0.133) | (0.196) | (0.180) |
|  |  |  |  |  |  |
| Wave Fixed Effects |  |  |  |  |  |
| Individual Fixed Effects |  |  |  |  |  |
|  |  |  |  |  |  |
| Observations | 5,261 | 26,826 | 15,858 | 27,978 | 19,967 |
| Number of individuals | 877 | 4,471 | 2,643 | 4,663 | 3,328 |
| NOTES: Each column reports results from a different regression, using the subsample of the corresponding socioeconomic group .Robust standard errors clustered at primary sampling unit in parentheses *** p<0.01, ** p<0.05, * p<0.1. ^†^Age groups are based on respondent age by April 2020. | | | | | |

Table A4- Difference-in-difference (DiD) results by socioeconomic group (II). GHQ-caseness

|  | Education level by wave 10 | | Household income by wave 10 | | Earning loss (April 2020) ^†^ | | Financial situation (April 2020)^‡^ | |
| --- | --- | --- | --- | --- | --- | --- | --- | --- |
| VARIABLES | A-level or lower | Higher A level | Below median | Above median | Yes | No | Bad | Good |
|  |  |  |  |  |  |  |  |  |
| DiD interactions |  |  |  |  |  |  |  |  |
| (base category: Wave Covid 1) |  |  |  |  |  |  |  |  |
| England x Wave 9 | -0.035 | 0.066 | 0.210 | -0.379 | -0.174 | 0.091 | -0.098 | 0.055 |
|  | (0.208) | (0.328) | (0.236) | (0.277) | (0.304) | (0.219) | (0.338) | (0.204) |
| England x Wave 10 | -0.174 | -0.039 | 0.068 | -0.154 | -0.567* | 0.164 | -0.334 | -0.013 |
|  | (0.288) | (0.299) | (0.282) | (0.307) | (0.312) | (0.259) | (0.488) | (0.200) |
| **England x Wave Covid 2** | -0.732** | 0.155 | -0.320 | -0.227 | -0.777** | -0.090 | -0.902** | -0.044 |
|  | (0.306) | (0.196) | (0.239) | (0.229) | (0.385) | (0.166) | (0.404) | (0.141) |
| England x Wave Covid 3 | -0.206 | -0.022 | -0.035 | -0.308 | -0.250 | -0.205 | -0.605 | -0.051 |
|  | (0.334) | (0.241) | (0.292) | (0.211) | (0.383) | (0.236) | (0.512) | (0.189) |
| England x Wave Covid 4 | -0.036 | 0.503** | 0.003 | -0.018 | -0.077 | -0.010 | -0.455 | 0.151 |
|  | (0.259) | (0.253) | (0.289) | (0.240) | (0.319) | (0.260) | (0.521) | (0.170) |
| Age | -0.015 | -0.063 | -0.023 | -0.023 | -0.059 | -0.019 | 0.116 | -0.092 |
|  | (0.087) | (0.114) | (0.092) | (0.082) | (0.123) | (0.076) | (0.144) | (0.071) |
| Living alone | 0.013 | -0.160 | -0.124 | -0.013 | -0.393* | 0.100 | -0.227 | -0.009 |
|  | (0.188) | (0.205) | (0.184) | (0.196) | (0.230) | (0.160) | (0.257) | (0.152) |
|  |  |  |  |  |  |  |  |  |
| Wave Fixed Effects | Yes | Yes | Yes | Yes | Yes | Yes | Yes | Yes |
| Individual Fixed Effects | Yes | Yes | Yes | Yes | Yes | Yes | Yes | Yes |
|  |  |  |  |  |  |  |  |  |
| Observations | 21,809 | 21,012 | 23,400 | 23,850 | 13,913 | 33,984 | 8,741 | 39,186 |
| Number of individuals | 3,635 | 3,502 | 3,900 | 3,975 | 2,319 | 5,664 | 1,457 | 6,531 |

NOTES: Each column reports results from a different regression, using the subsample of the corresponding socioeconomic group. Robust standard errors clustered at primary sampling unit in parentheses *** p<0.01, ** p<0.05, * p<0.1. ^†^ Based on the question “Is your household is now earning less than in January/February 2020?”. ^‡^ Based on the question “How well would you say you yourself are managing financially these days?”; those who responded “living comfortably” or “doing alright” were classified as Good financial situation, whereas those who responded “Just about getting by”, “Finding it quite difficult” or “Finding it very difficult” were classified as Bad financial situation.

Figure A1 - 7-day rolling average of COVID-19 cases in England vs Scotland


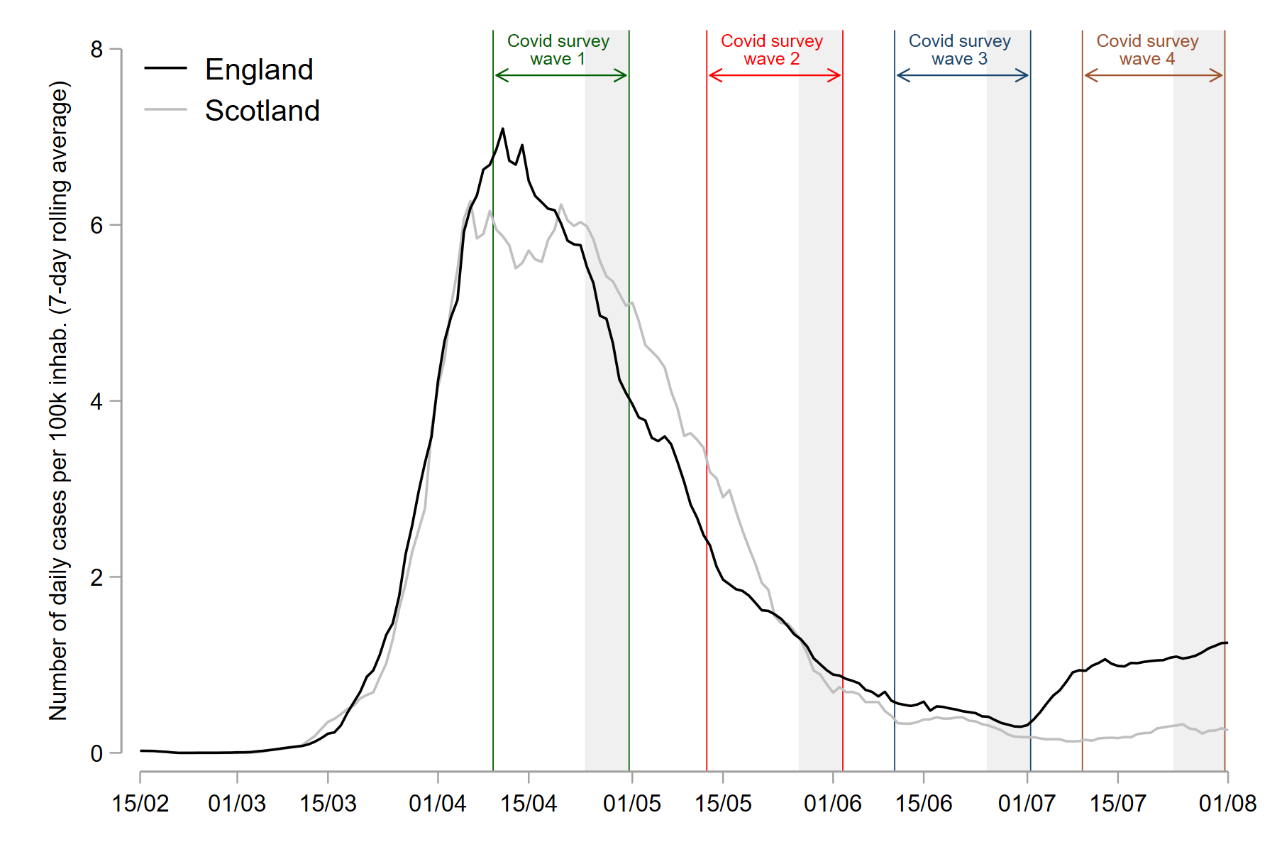


NOTES: Covid-19 cases are based on the publish date and reported as a 7-day rolling average. Data source: UK government (<https://coronavirus.data.gov.uk/details/download>)

Figure A2- Mobility in England vs Scotland


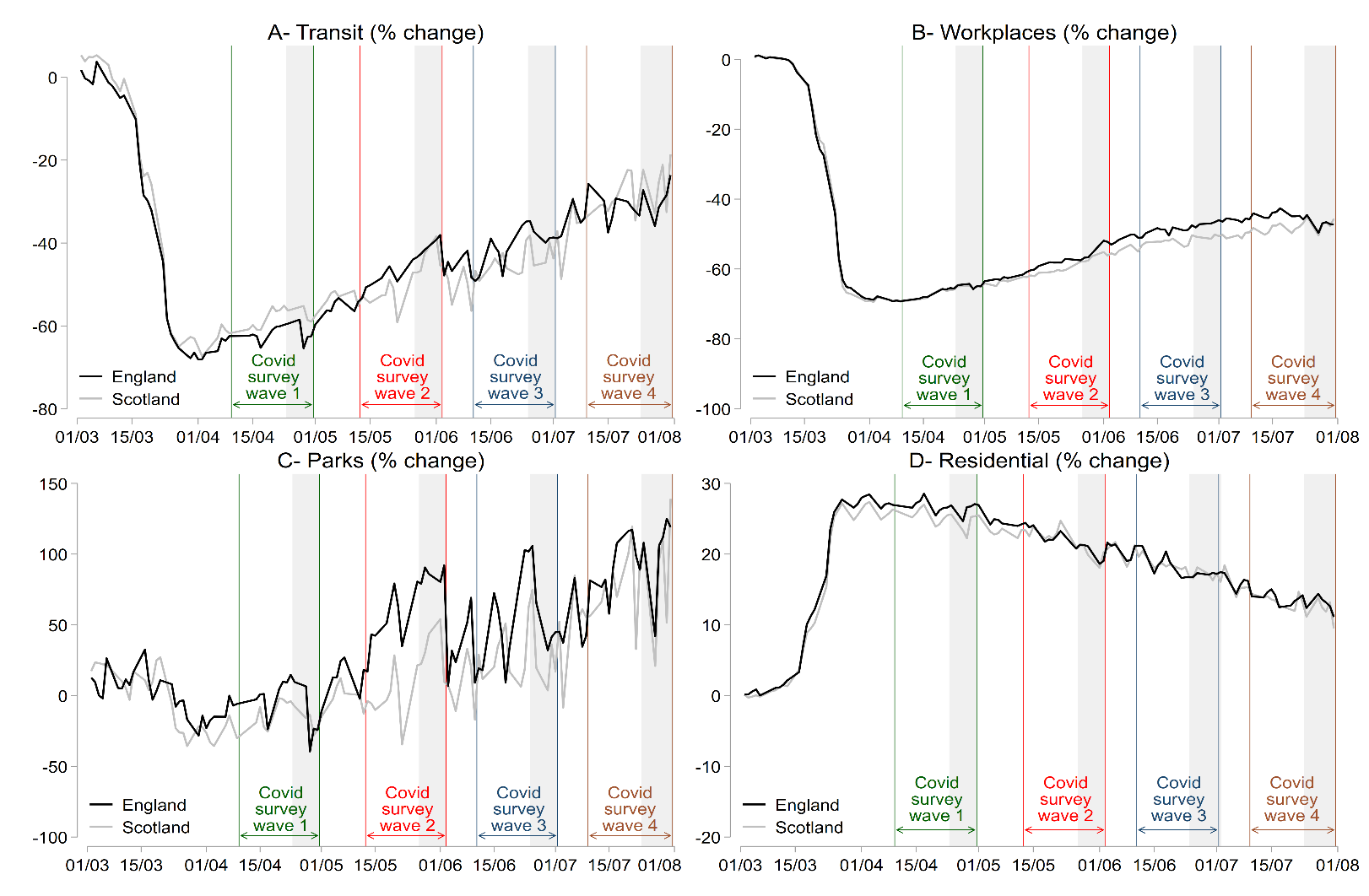


NOTES: Figures report the percentage change in mobility for each dimension with respect to the median value for the corresponding day of the week during the period 3 January 2020 – 6 February 2020. We excluded weekend days and bank holidays to avoid fluctuations. Shaded areas represent the fieldwork days for each UKHLS survey wave. The areas between the colourful vertical lines represent the reference period for each survey wave regarding the mental health questions. Google mobility data is based on Google Account users who have opted in to Location History. More information about this dataset can be found at <https://support.google.com/covid19-mobility/answer/9824897?hl=en&ref_topic=9822927>

Figure A3- Mean GHQ-caseness by age group

| Subsample A- Young adults (16-34) |
| --- |
| 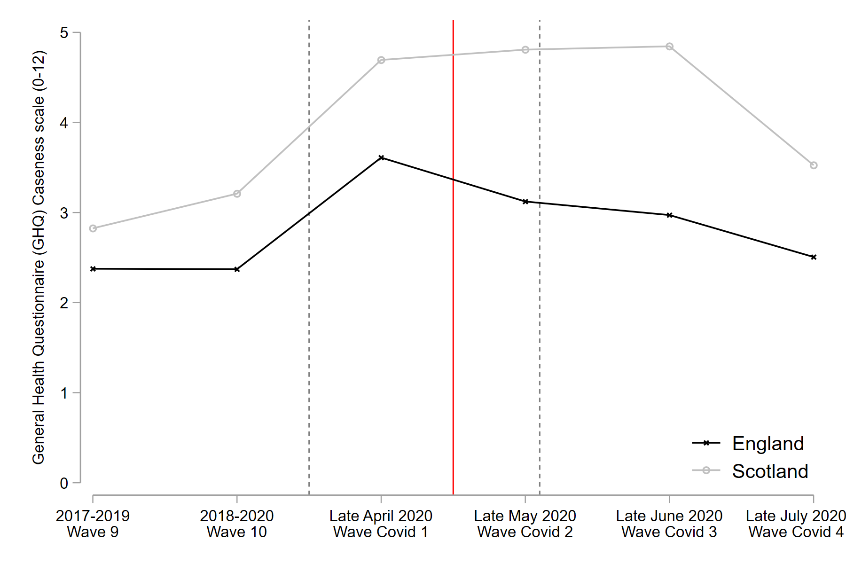 |
| Subsample B- Adults (35-64) |
| 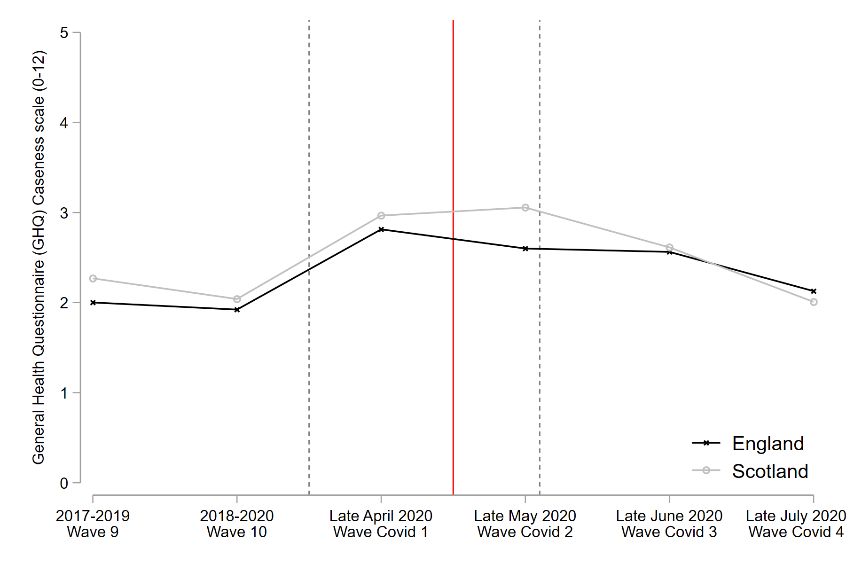 |
| Subsample C- Elderly (65+) |
| 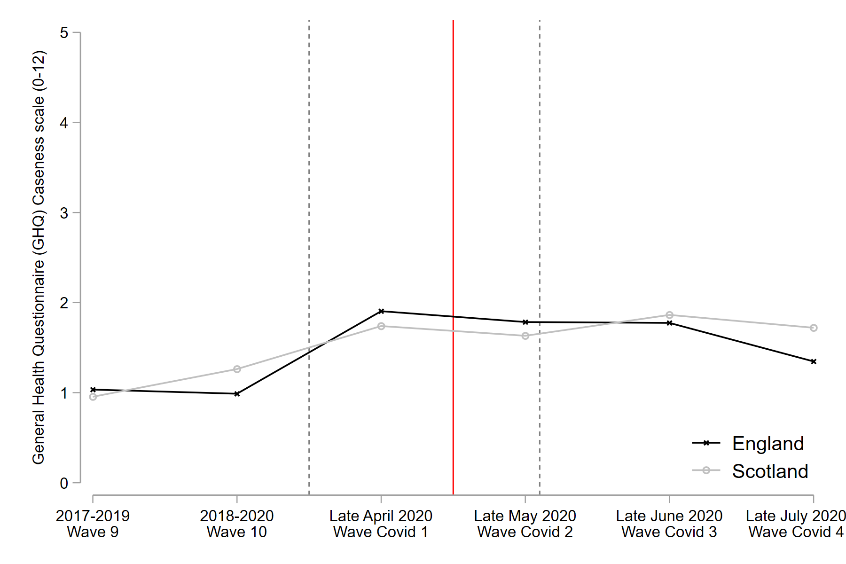 |

NOTES: Weighted mean GHQ-caseness scale (0-12) by nation and wave for each corresponding subsample. The age of each subsample is calculated as of April 2020. Number of individuals per subsample: Subsample A (Young adults), n=1,083 (England: 985, Scotland: 98). Subsample B (Adults), n=5,139 (England: 4,608; Scotland: 531). Subsample C (Elderly), n=2,857 (England: 2,571; Scotland: 286).

Figure A4- Mean GHQ-caseness by gender.

| Subsample A- Women |
| --- |
| 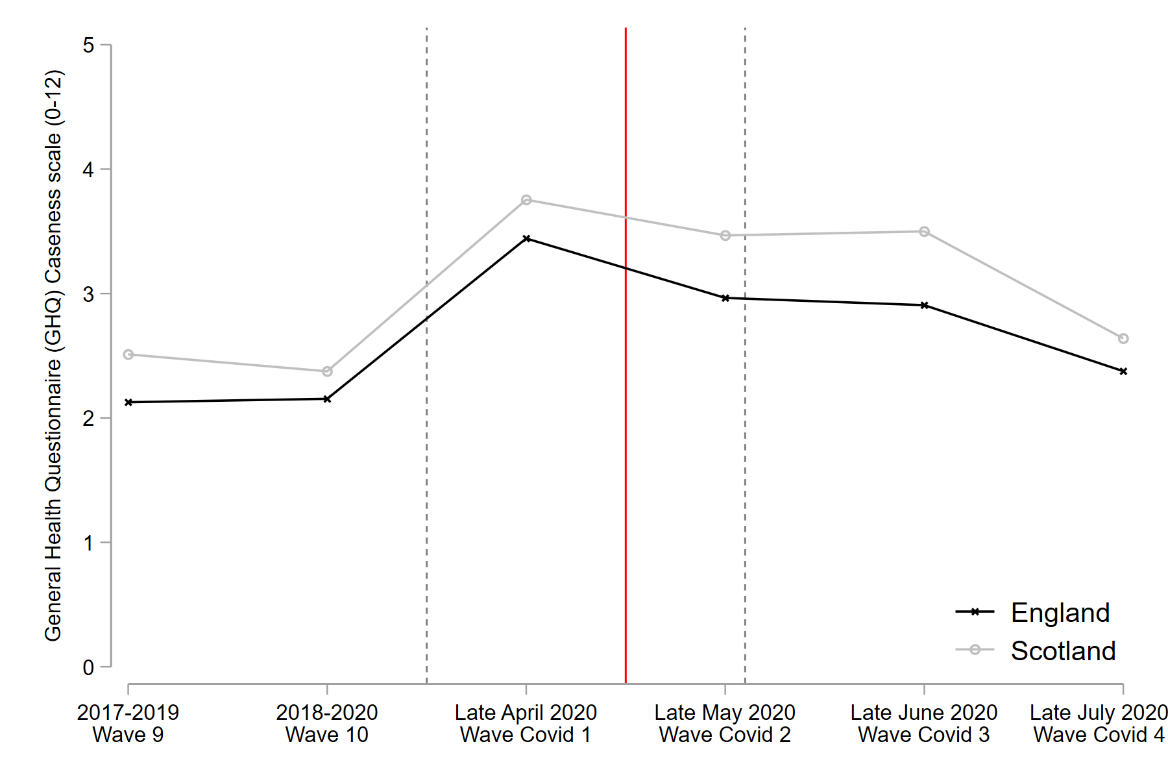 |
| Subsample B- Men |
| 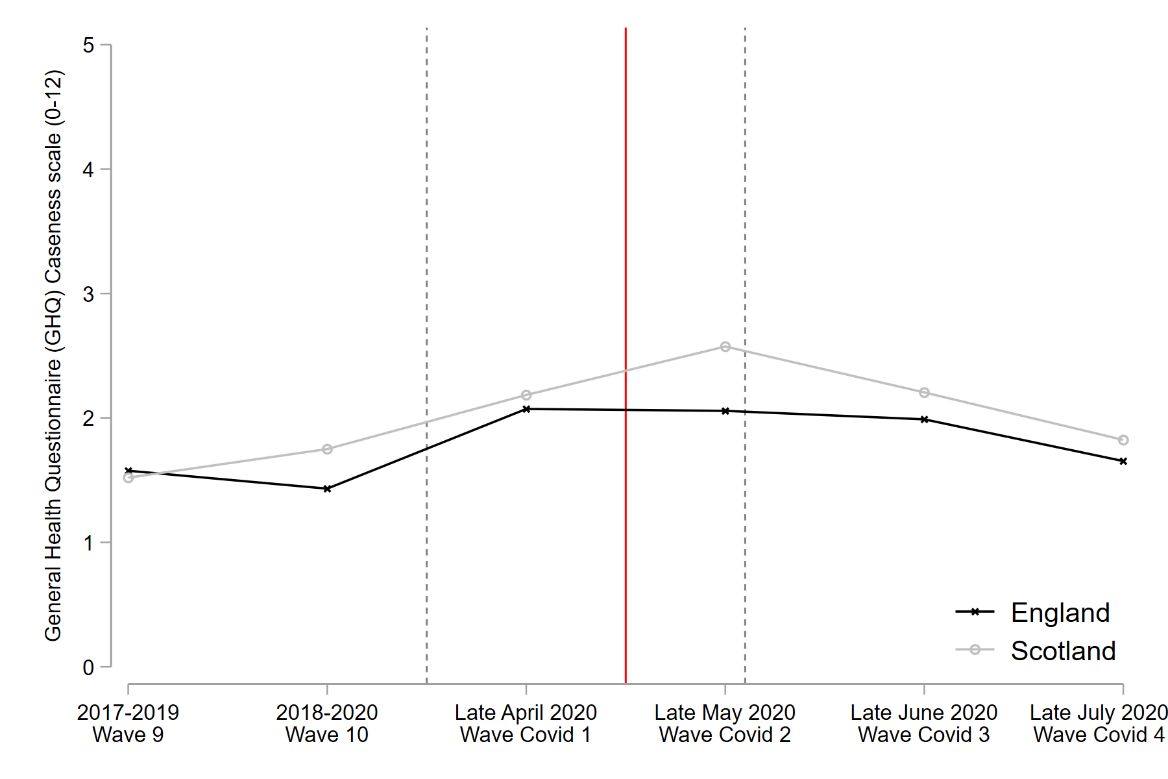 |

NOTES: Weighted mean GHQ-caseness scale (0-12) by nation and wave for each corresponding subsample. Number of individuals per subsample: Subsample A (Women), n=5,308 (England: 4,769, Scotland: 539). Subsample B (Men), n=3,771 (England: 3,395; Scotland: 376).

Figure A5- Mean GHQ-caseness by educational level

| Subsample A- Education- A-level or lower |
| --- |
| 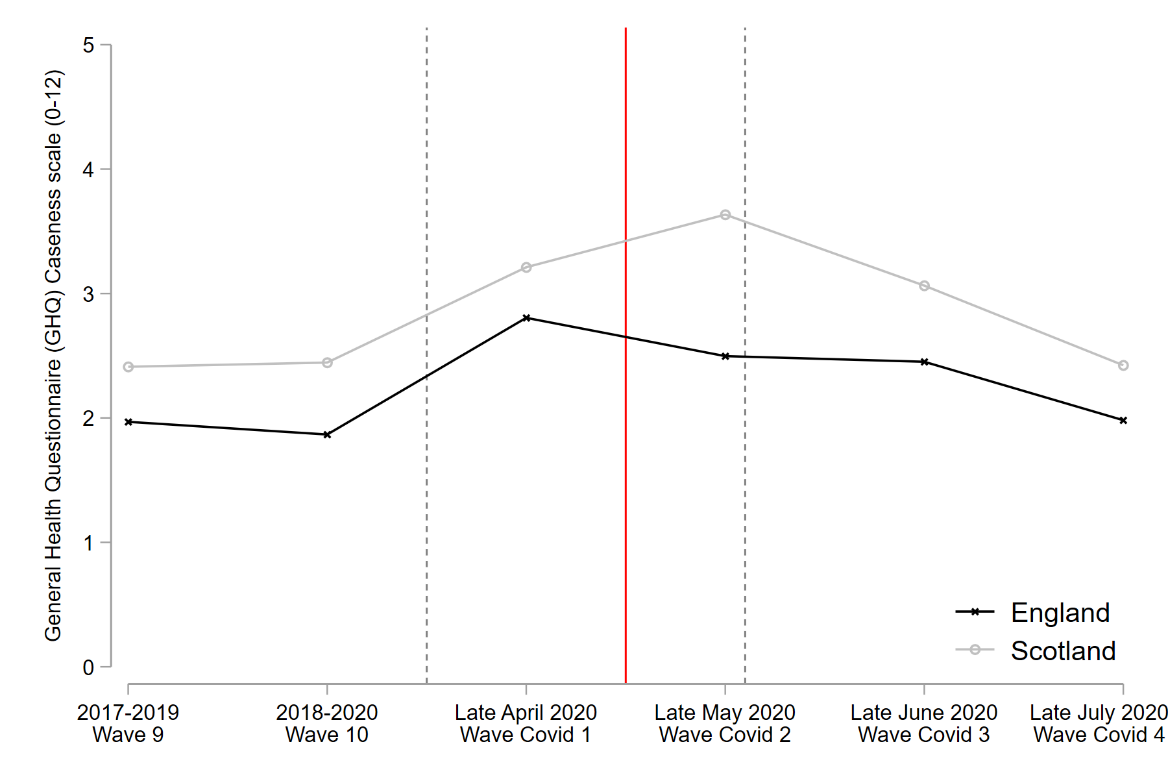 |
| Subsample B- Education- Higher than A level |
| 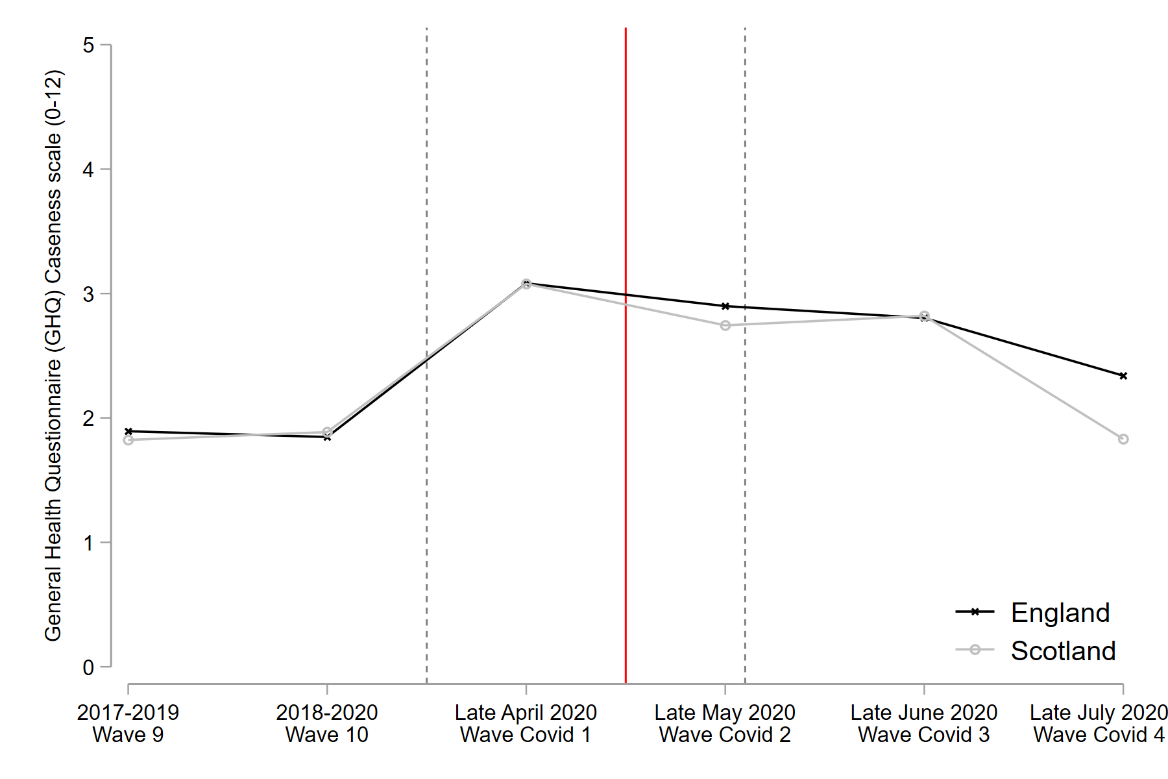 |

NOTES: Weighted mean GHQ-caseness scale (0-12) by nation and wave for each corresponding subsample. Number of individuals per subsample: Subsample A (A-level or lower), n=4,115 (England: 3,760 Scotland: 355). Subsample B (Higher than A level), n=4,001 (England: 3,669; Scotland: 332).

Figure A6- Mean GHQ-caseness by household income

| Subsample A- Household income at wave 10- Below median |
| --- |
| 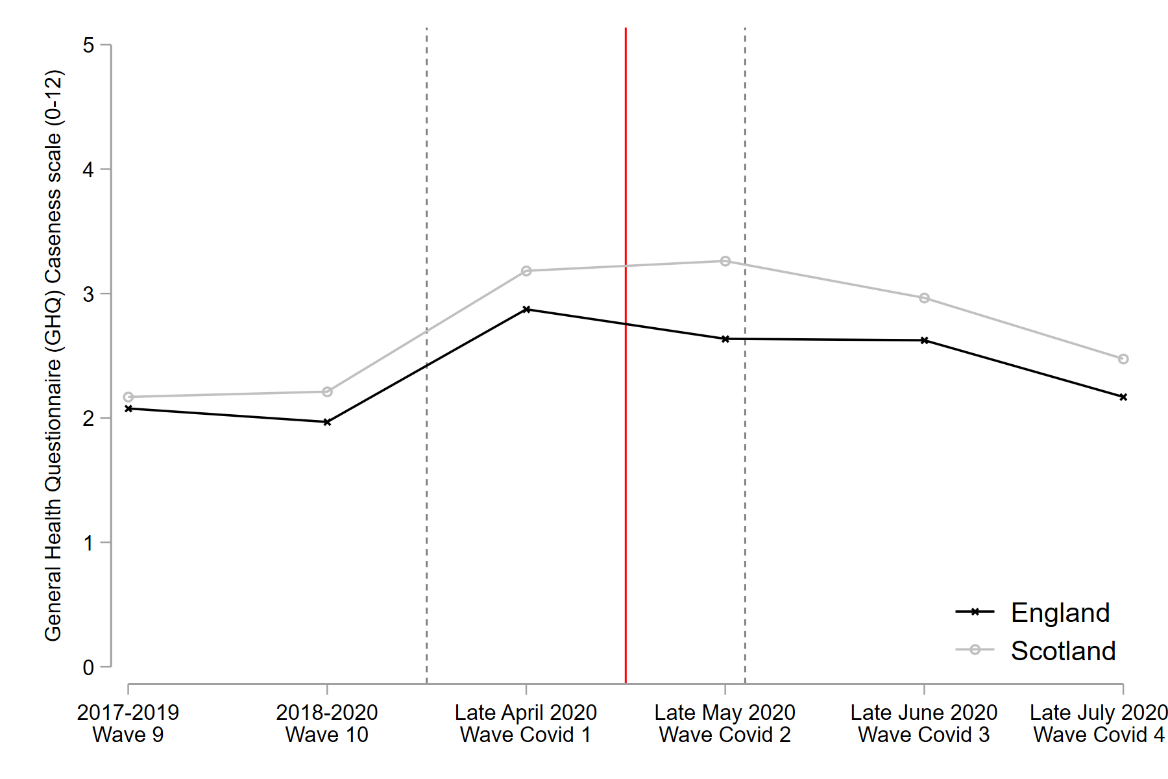 |
| Subsample B- Household income at wave 10- Above median |
| 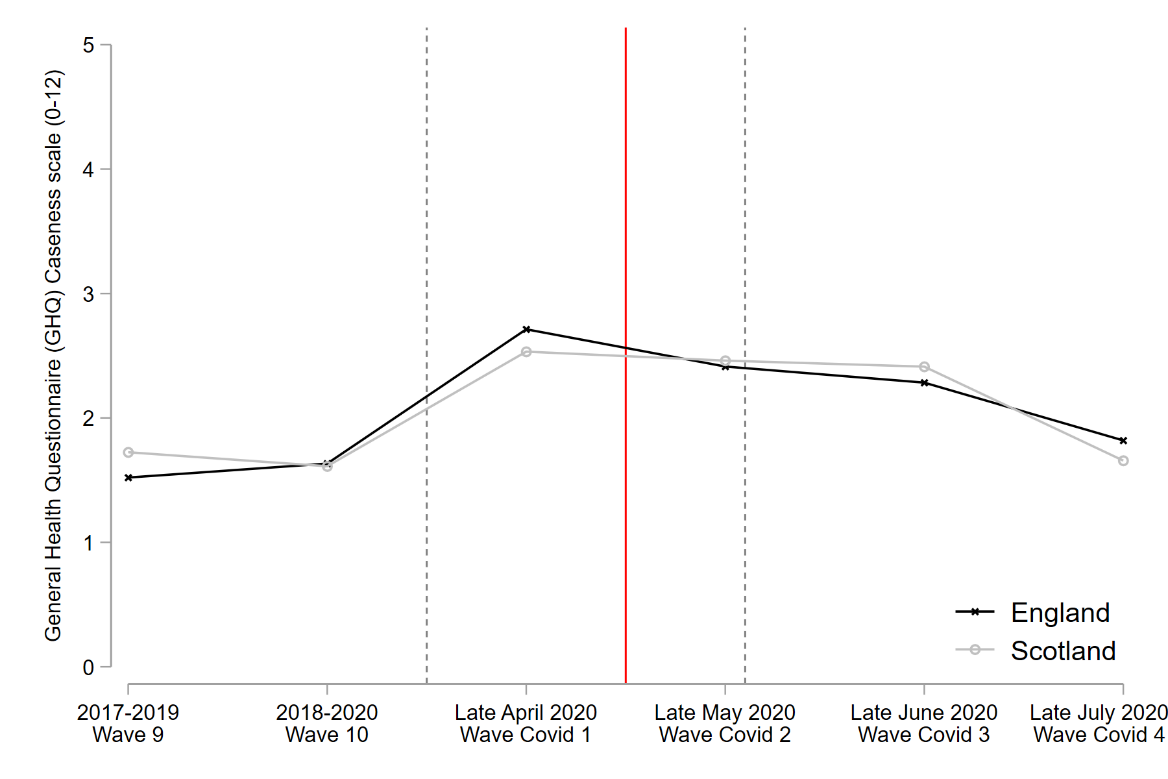 |

NOTES: Weighted mean GHQ-caseness scale (0-12) by nation and wave for each corresponding subsample. Number of individuals per subsample: Subsample A (Household income below median), n=4,469 (England: 3,987 Scotland: 482). Subsample B (Higher than A level), n=4,469 (England: 4,047; Scotland: 422).

Figure A7- Mean GHQ-caseness by financial situation

| Subsample A- Bad financial situation: “getting by or difficult” by April 2020 |  |
| --- | --- |
| 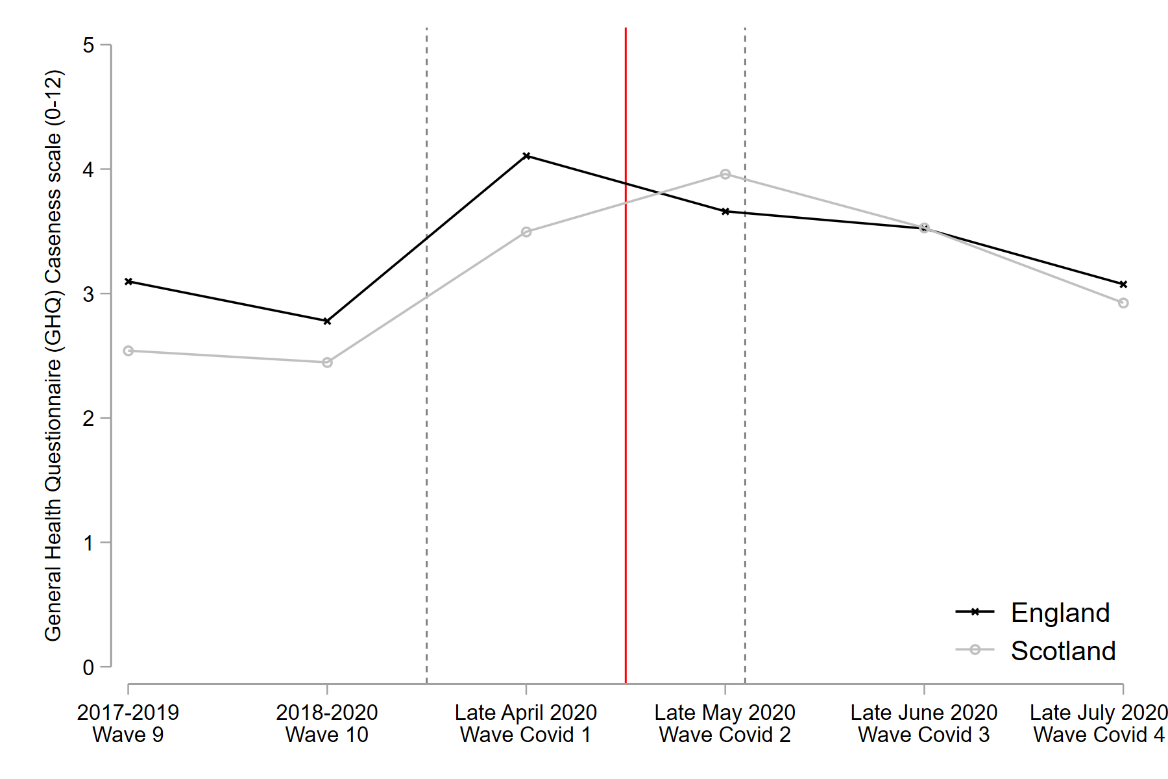 | |
| Subsample B- Good financial situation: “doing alright or living comfortably” by April 2020 |  |
| 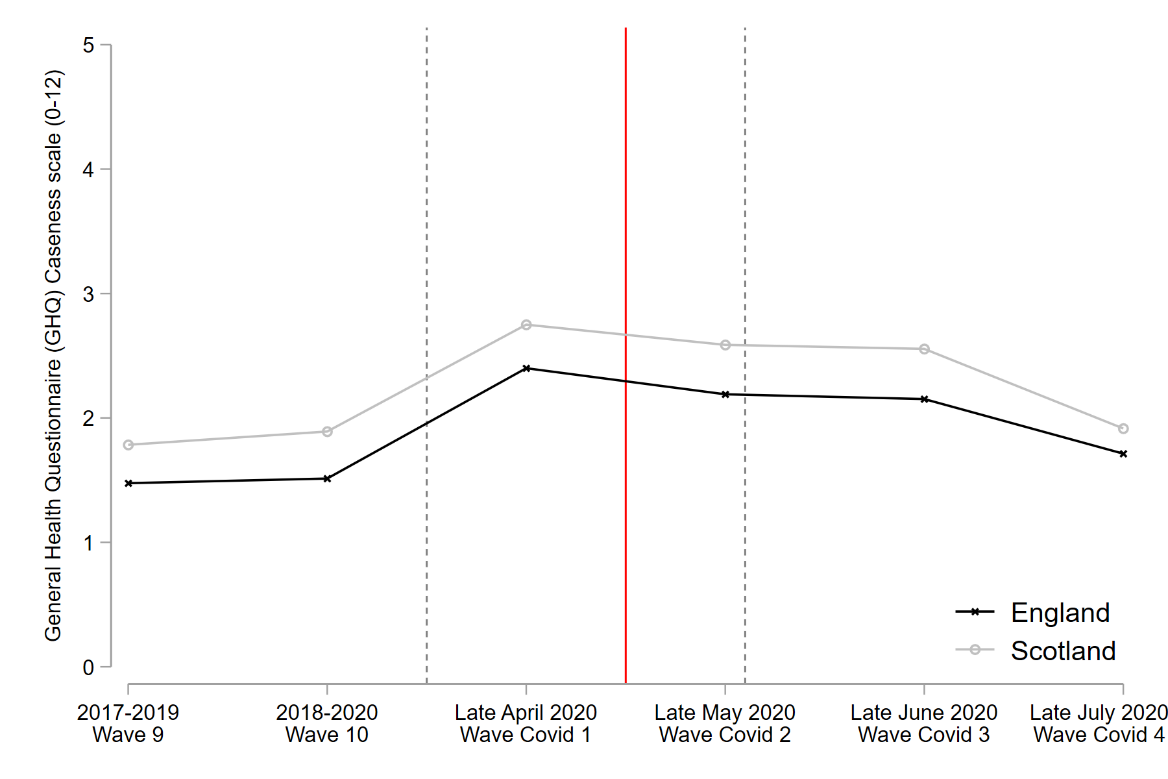 |  |

NOTES: Weighted mean GHQ-caseness scale (0-12) by nation and wave for each corresponding subsample. Number of individuals per subsample: Subsample A (Bad financial situation), n=1,701 (England: 1,524 Scotland: 177). Subsample B (Good financial situation), n=7,375 (England: 6,637; Scotland: 738).

Figure A8- Mean GHQ-caseness by household earning loss April 2020

| Subsample A- Household earning loss in April with respect to Jan/Feb 2020 |
| --- |
| 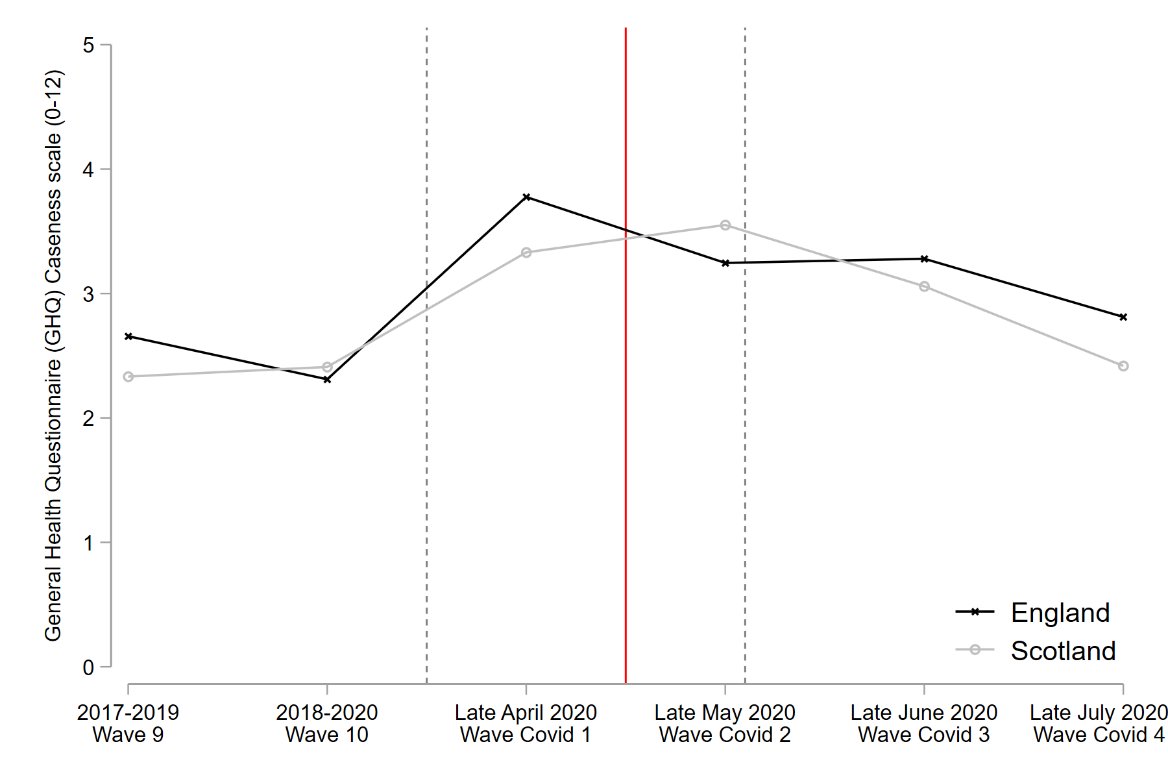 |
| Subsample B- No household earning loss in April with respect to Jan/Feb 2020 |
| 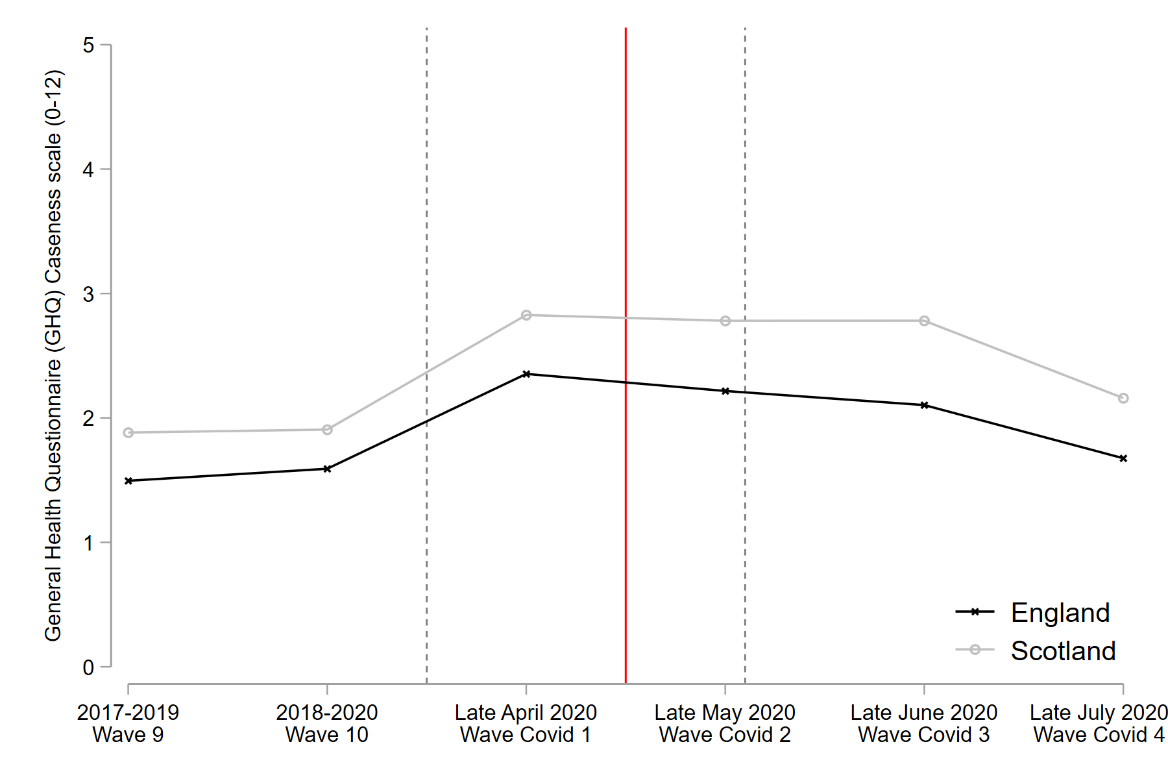 |

NOTES: Weighted mean GHQ-caseness scale (0-12) by nation and wave for each corresponding subsample. Number of individuals per subsample: Subsample A (Earning loss), n=2,701 (England: 2,438 Scotland: 263). Subsample B (No earning loss), n=6,370 (England: 5,719; Scotland: 651).

Figure A9- Probability of employment by nation and time.


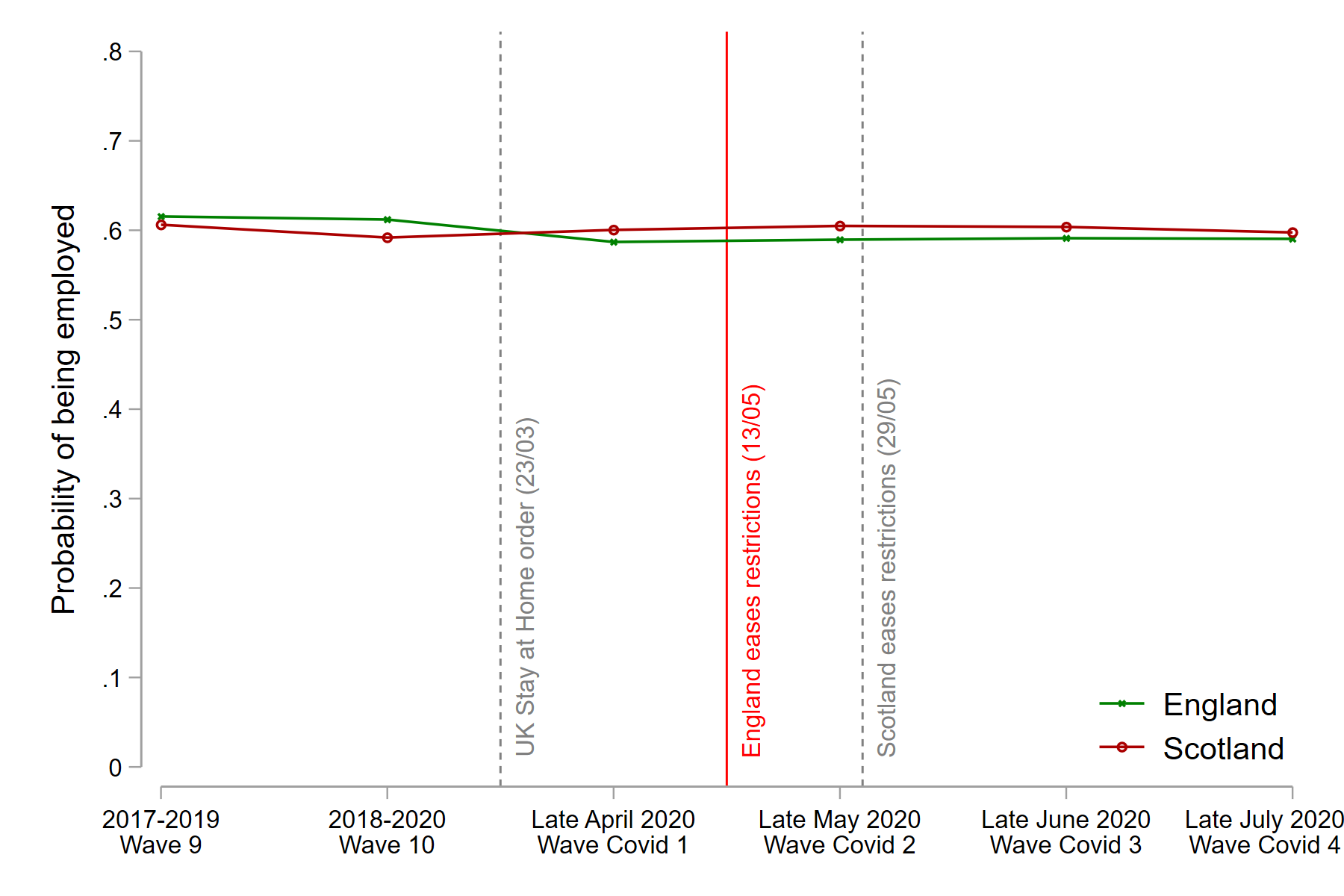


NOTES: This figure reports the weighted probability of being employed over nation and UKHLS wave.

Table A5- Difference-in-difference (DiD) results. Mediating analysis. GHQ-caseness

|  |  |  |  |
| --- | --- | --- | --- |
| VARIABLES | (1) | (2) | (3) |
|  |  |  |  |
| DiD interactions |  |  |  |
| (base category: Wave Covid 1) |  |  |  |
| England x Wave 9 | 0.0187 | -0.0846 | 0.0236 |
|  | (0.162) | (0.192) | (0.162) |
| England x Wave 10 | -0.0697 | -0.136 | -0.0409 |
|  | (0.197) | (0.200) | (0.196) |
| **England x Wave Covid 2** | -0.311* | -0.339** | -0.313* |
|  | (0.169) | (0.169) | (0.169) |
| England x Wave Covid 3 | -0.211 | -0.267 | -0.213 |
|  | (0.206) | (0.209) | (0.206) |
| England x Wave Covid 4 | -0.0237 | -0.105 | -0.0237 |
|  | (0.208) | (0.206) | (0.209) |
| Age | -0.0342 | -0.0156 | -0.0346 |
|  | (0.065) | (0.063) | (0.065) |
| Living alone | -0.0812 | -0.0581 | -0.0814 |
|  | (0.136) | (0.129) | (0.136) |
| Feeling lonely |  | 1.311*** |  |
|  |  | (0.099) |  |
| Employed |  |  | -0.299** |
|  |  |  | (0.131) |
|  |  |  |  |
| Wave Fixed Effects | Yes | Yes | Yes |
| Individual Fixed Effects | Yes | Yes | Yes |
|  |  |  |  |
| Observations | 47,945 | 47,934 | 47,897 |
| Number of individuals | 7,991 | 7,991 | 7,991 |

NOTES: Each column reports results from a different regression, where the dependent variable is the GHQ-caseness (0-12). Robust standard errors clustered at primary sampling unit in parentheses. *** p<0.01, ** p<0.05, * p<0.1. Note that the difference in observations between the final balanced sample (n=9,079) and the sample used in the regressions (n=7,991) is due to some survey respondents (1,088) having zero weights by sampling design (Kaminska & Lynn, 2019).

Table A6- Difference-in-difference (DiD) results. GHQ-likert

|  |  |  |  |
| --- | --- | --- | --- |
| VARIABLES | (1) | (2) | (3) |
|  |  |  |  |
| DiD interactions |  |  |  |
| (base category: Wave Covid 1) |  |  |  |
| England x Wave 9 | 0.483 | 0.493 | 0.482 |
|  | (0.324) | (0.326) | (0.325) |
| England x Wave 10 | -0.0674 | -0.0536 | -0.0583 |
|  | (0.346) | (0.344) | (0.344) |
| **England x Wave Covid 2** | -0.438* | -0.439* | -0.442* |
|  | (0.257) | (0.258) | (0.258) |
| England x Wave Covid 3 | -0.00599 | -0.00763 | -0.00977 |
|  | (0.311) | (0.311) | (0.311) |
| England x Wave Covid 4 | 0.357 | 0.358 | 0.356 |
|  | (0.288) | (0.288) | (0.288) |
| Age |  | -0.0748 | -0.0744 |
|  |  | (0.123) | (0.123) |
| Living alone |  |  | -0.216 |
|  |  |  | (0.227) |
|  |  |  |  |
| Wave Fixed Effects | Yes | Yes | Yes |
| Individual Fixed Effects | Yes | Yes | Yes |
|  |  |  |  |
| Observations | 47,946 | 47,945 | 47,945 |
| Number of individuals | 7,991 | 7,991 | 7,991 |

NOTES: Each column reports results from a different regression, where the dependent variable is the GHQ-likert scale (0-36). Robust standard errors clustered at primary sampling unit in parenthesis. *** p<0.01, ** p<0.05, * p<0.1. Note that the difference in observations between the final balanced sample (n=9,079) and the sample used in the regressions (n=7,991) is due to some survey respondents (1,088) having zero weights by sampling design (Kaminska & Lynn, 2019).

Table A7- Difference-in-difference (DiD) results by socioeconomic group (I). GHQ-likert

|  | Age^†^ | | | Sex | |
| --- | --- | --- | --- | --- | --- |
| VARIABLES | 16-34 | 35-64 | 65+ | Women | Men |
|  |  |  |  |  |  |
| DiD interactions |  |  |  |  |  |
| (base category: Wave Covid 1) |  |  |  |  |  |
| England x Wave 9 | 1.446 | 0.001 | 0.817 | 0.177 | 0.841 |
|  | (0.887) | (0.371) | (0.664) | (0.421) | (0.532) |
| England x Wave 10 | 0.346 | 0.258 | -0.771 | 0.102 | -0.174 |
|  | (1.021) | (0.432) | (0.487) | (0.450) | (0.524) |
| **England x Wave Covid 2** | -0.856 | -0.375 | -0.158 | -0.362 | -0.487 |
|  | (0.783) | (0.317) | (0.273) | (0.376) | (0.365) |
| England x Wave Covid 3 | -0.683 | 0.254 | 0.038 | -0.323 | 0.378 |
|  | (1.038) | (0.383) | (0.247) | (0.459) | (0.427) |
| England x Wave Covid 4 | 0.363 | 0.701* | -0.231 | 0.169 | 0.617* |
|  | (0.898) | (0.367) | (0.249) | (0.451) | (0.348) |
| Age | -0.039 | -0.019 | -0.162 | -0.108 | -0.033 |
|  | (0.374) | (0.131) | (0.116) | (0.143) | (0.200) |
| Living alone | 0.347 | -0.669** | 0.191 | 0.024 | -0.513 |
|  | (0.558) | (0.279) | (0.203) | (0.319) | (0.318) |
|  |  |  |  |  |  |
| Wave Fixed Effects | Yes | Yes | Yes | Yes | Yes |
| Individual Fixed Effects | Yes | Yes | Yes | Yes | Yes |
|  |  |  |  |  |  |
| Number of observations | 5,261 | 26,826 | 15,858 | 27,978 | 19,967 |
| Number of individuals | 877 | 4,471 | 2,643 | 4,663 | 3,328 |
| NOTES: Each column reports results from a different regression, using the subsample of the corresponding socioeconomic group where the dependent variable is the GHQ-likert scale (0-36). Robust standard errors clustered at primary sampling unit in parentheses *** p<0.01, ** p<0.05, * p<0.1. ^†^Age groups are based on respondent age by April 2020. | | | | | |

Table A8- Difference-in-difference (DiD) results by socioeconomic group (II). GHQ-likert

|  | Education level by wave 10 | | Household income by wave 10 | | Earning loss (April 2020) ^†^ | | Financial situation (April 2020)^‡^ | |
| --- | --- | --- | --- | --- | --- | --- | --- | --- |
| VARIABLES | A-level or lower | Higher A level | Below median | Above median | Yes | No | Bad | Good |
|  |  |  |  |  |  |  |  |  |
| DiD interactions |  |  |  |  |  |  |  |  |
| (base category: Wave Covid 1) |  |  |  |  |  |  |  |  |
| England x Wave 9 | -0.108 | 0.560 | 0.840* | -0.177 | -0.003 | 0.700 | 0.425 | 0.408 |
|  | (0.383) | (0.571) | (0.470) | (0.441) | (0.469) | (0.441) | (0.679) | (0.349) |
| England x Wave 10 | -0.423 | 0.328 | -0.064 | 0.223 | -0.844 | 0.327 | -0.753 | 0.136 |
|  | (0.470) | (0.644) | (0.466) | (0.557) | (0.602) | (0.412) | (0.827) | (0.335) |
| **England x Wave Covid 2** | -1.159*** | 0.027 | -0.452 | -0.245 | -0.999** | -0.175 | -1.196** | -0.137 |
|  | (0.394) | (0.309) | (0.378) | (0.309) | (0.471) | (0.305) | (0.574) | (0.259) |
| England x Wave Covid 3 | -0.363 | 0.144 | 0.291 | -0.181 | -0.136 | 0.040 | -0.235 | 0.029 |
|  | (0.581) | (0.373) | (0.450) | (0.292) | (0.598) | (0.333) | (0.671) | (0.344) |
| England x Wave Covid 4 | -0.012 | 0.901* | 0.489 | 0.277 | 0.056 | 0.491 | 0.141 | 0.367 |
|  | (0.431) | (0.464) | (0.380) | (0.385) | (0.560) | (0.319) | (0.563) | (0.312) |
| Age | 0.031 | -0.209 | -0.091 | -0.007 | -0.009 | -0.112 | 0.165 | -0.161 |
|  | (0.190) | (0.156) | (0.184) | (0.130) | (0.270) | (0.122) | (0.351) | (0.106) |
| Living alone | -0.131 | -0.287 | -0.257 | -0.168 | -0.746* | 0.102 | -0.678* | 0.003 |
|  | (0.315) | (0.367) | (0.318) | (0.279) | (0.387) | (0.268) | (0.386) | (0.259) |
|  |  |  |  |  |  |  |  |  |
| Wave Fixed Effects | Yes | Yes | Yes | Yes | Yes | Yes | Yes | Yes |
| Individual Fixed Effects | Yes | Yes | Yes | Yes | Yes | Yes | Yes | Yes |
|  |  |  |  |  |  |  |  |  |
| Observations | 21,809 | 21,012 | 23,400 | 23,850 | 13,913 | 33,984 | 8,741 | 39,186 |
| Number of individuals | 3,635 | 3,502 | 3,900 | 3,975 | 2,319 | 5,664 | 1,457 | 6,531 |

NOTES: Each column reports results from a different regression, using the subsample of the corresponding socioeconomic group, where the dependent variable is the GHQ-likert scale (0-36). Robust standard errors clustered at primary sampling unit in parentheses *** p<0.01, ** p<0.05, * p<0.1. ^†^ Based on the question “Is your household is now earning less than in January/February 2020?”. ^‡^ Based on the question “How well would you say you yourself are managing financially these days?”; those who responded “living comfortably” or “doing alright” were classified as Good financial situation, whereas those who responded “Just about getting by”, “Finding it quite difficult” or “Finding it very difficult” were classified as Bad financial situationES: Robust standard errors in parentheses *** p<0.01, ** p<0.05, * p<0.1.

Table A9- Difference-in-difference (DiD) results. GHQ-binary

|  |  |  |  |
| --- | --- | --- | --- |
| VARIABLES | (1) | (2) | (3) |
|  |  |  |  |
| DiD interactions |  |  |  |
| (base category: Wave Covid 1) |  |  |  |
| England x Wave 9 | 0.0137 | 0.0142 | 0.0152 |
|  | (0.026) | (0.026) | (0.026) |
| England x Wave 10 | -0.0441 | -0.0430 | -0.0426 |
|  | (0.061) | (0.059) | (0.059) |
| **England x Wave Covid 2** | -0.0663*** | -0.0663*** | -0.0661*** |
|  | (0.021) | (0.021) | (0.021) |
| England x Wave Covid 3 | -0.0206 | -0.0207 | -0.0205 |
|  | (0.031) | (0.031) | (0.031) |
| England x Wave Covid 4 | -0.00909 | -0.00905 | -0.00885 |
|  | (0.026) | (0.026) | (0.026) |
| Age |  | -0.00360 | -0.00363 |
|  |  | (0.009) | (0.009) |
| Living alone |  |  | 0.0191 |
|  |  |  | (0.022) |
|  |  |  |  |
| Wave Fixed Effects | Yes | Yes | Yes |
| Individual Fixed Effects | Yes | Yes | Yes |
|  |  |  |  |
| Observations | 47,946 | 47,945 | 47,945 |
| Number of individuals | 7,991 | 7,991 | 7,991 |

NOTES: Each column reports results from a different regression, where the dependent variable is the dummy GHQ-binary. Robust standard errors clustered at primary sampling unit in parentheses. *** p<0.01, ** p<0.05, * p<0.1 Note that the difference in observations between the final balanced sample (n=9,079) and the sample used in the regressions (n=7,991) is due to some survey respondents (1,088) having zero weights by sampling design (Kaminska & Lynn, 2019).

Table A10- Difference-in-difference (DiD) results by socioeconomic group (I). GHQ-binary

|  | Age^†^ | | | Sex | |
| --- | --- | --- | --- | --- | --- |
| VARIABLES | 16-34 | 35-64 | 65+ | Women | Men |
|  |  |  |  |  |  |
| DiD interactions |  |  |  |  |  |
| (base category: Wave Covid 1) |  |  |  |  |  |
| England x Wave 9 | 0.056 | 0.017 | -0.022 | 0.030 | 0.001 |
|  | (0.078) | (0.032) | (0.037) | (0.039) | (0.030) |
| England x Wave 10 | -0.014 | 0.033 | -0.204 | 0.013 | -0.097 |
|  | (0.090) | (0.033) | (0.167) | (0.047) | (0.102) |
| **England x Wave Covid 2** | -0.074 | -0.035 | -0.104*** | -0.060** | -0.070** |
|  | (0.077) | (0.026) | (0.029) | (0.029) | (0.033) |
| England x Wave Covid 3 | -0.060 | -0.001 | -0.016 | -0.053 | 0.016 |
|  | (0.081) | (0.044) | (0.029) | (0.047) | (0.040) |
| England x Wave Covid 4 | 0.014 | 0.010 | -0.061 | 0.009 | -0.025 |
|  | (0.088) | (0.027) | (0.042) | (0.037) | (0.036) |
| Age | -0.005 | 0.005 | -0.022 | 0.003 | -0.010 |
|  | (0.017) | (0.009) | (0.019) | (0.011) | (0.013) |
| Living alone | 0.086** | -0.033 | 0.029 | 0.044* | -0.013 |
|  | (0.044) | (0.029) | (0.019) | (0.026) | (0.034) |
|  |  |  |  |  |  |
| Wave Fixed Effects | Yes | Yes | Yes | Yes | Yes |
| Individual Fixed Effects | Yes | Yes | Yes | Yes | Yes |
|  |  |  |  |  |  |
| Observations | 5,261 | 26,826 | 15,858 | 27,978 | 19,967 |
| Number of individuals | 877 | 4,471 | 2,643 | 4,663 | 3,328 |
| NOTES: Each column reports results from a different regression, using the subsample of the corresponding socioeconomic group, where the dependent variable is the dummy GHQ-binary. Robust standard errors clustered at primary sampling unit in parentheses *** p<0.01, ** p<0.05, * p<0.1. ^†^Age groups are based on respondent age by April 2020 | | | | | |

Table A11- Difference-in-difference (DiD) results by socioeconomic group (I). GHQ-binary

|  | Education level by wave 10 | | Household income by wave 10 | | Earning loss (April 2020) ^†^ | | Financial situation (April 2020)^‡^ | |
| --- | --- | --- | --- | --- | --- | --- | --- | --- |
| VARIABLES | A-level or lower | Higher A level | Below median | Above median | Yes | No | Bad | Good |
|  |  |  |  |  |  |  |  |  |
| DiD interactions |  |  |  |  |  |  |  |  |
| (base category: Wave Covid 1) |  |  |  |  |  |  |  |  |
| England x Wave 9 | -0.006 | 0.049 | 0.024 | -0.011 | -0.045 | 0.043 | -0.086* | 0.054* |
|  | (0.039) | (0.040) | (0.035) | (0.031) | (0.041) | (0.032) | (0.049) | (0.028) |
| England x Wave 10 | -0.007 | 0.012 | -0.068 | 0.002 | -0.053 | -0.040 | -0.204 | 0.026 |
|  | (0.045) | (0.041) | (0.087) | (0.033) | (0.049) | (0.084) | (0.151) | (0.029) |
| **England x Wave Covid 2** | -0.087** | -0.014 | -0.085*** | -0.022 | -0.094** | -0.054** | -0.150*** | -0.034* |
|  | (0.038) | (0.035) | (0.030) | (0.025) | (0.038) | (0.026) | (0.051) | (0.019) |
| England x Wave Covid 3 | -0.006 | -0.013 | -0.017 | 0.016 | -0.048 | -0.009 | -0.079 | 0.002 |
|  | (0.049) | (0.065) | (0.044) | (0.028) | (0.070) | (0.028) | (0.081) | (0.026) |
| England x Wave Covid 4 | -0.040 | 0.077* | -0.013 | 0.012 | -0.033 | 0.001 | -0.078 | 0.017 |
|  | (0.042) | (0.041) | (0.038) | (0.028) | (0.047) | (0.031) | (0.063) | (0.024) |
| Age | 0.007 | -0.014 | -0.005 | -0.001 | -0.020 | 0.005 | -0.015 | 0.001 |
|  | (0.010) | (0.011) | (0.013) | (0.010) | (0.013) | (0.011) | (0.022) | (0.008) |
| Living alone | 0.021 | 0.021 | 0.017 | 0.016 | -0.003 | 0.030 | -0.024 | 0.037* |
|  | (0.034) | (0.023) | (0.031) | (0.022) | (0.045) | (0.020) | (0.047) | (0.020) |
| Employed | 0.009 | -0.022 | -0.000 | 0.012 | 0.010 | -0.008 | 0.014 | -0.008 |
|  | (0.019) | (0.033) | (0.022) | (0.023) | (0.028) | (0.018) | (0.036) | (0.016) |
|  |  |  |  |  |  |  |  |  |
| Wave Fixed Effects | Yes | Yes | Yes | Yes | Yes | Yes | Yes | Yes |
| Individual Fixed Effects | Yes | Yes | Yes | Yes | Yes | Yes | Yes | Yes |
|  |  |  |  |  |  |  |  |  |
| Observations | 21,809 | 21,012 | 23,400 | 23,850 | 13,913 | 33,984 | 8,741 | 39,186 |
| Number of individuals | 3,635 | 3,502 | 3,900 | 3,975 | 2,319 | 5,664 | 1,457 | 6,531 |

NOTES: Each column reports results from a different regression, using the subsample of the corresponding socioeconomic group where the dependent variable is the dummy GHQ-binary. Robust standard errors clustered at primary sampling unit in parentheses *** p<0.01, ** p<0.05, * p<0.1.^†^ Based on the question “Is your household is now earning less than in January/February 2020?”. ^‡^ Based on the question “How well would you say you yourself are managing financially these days?”; those who responded “living comfortably” or “doing alright” were classified as Good financial situation, whereas those who responded “Just about getting by”, “Finding it quite difficult” or “Finding it very difficult” were classified as Bad financial situation.

Table A12- Difference-in-difference (DiD) results. GHQ-binary2

|  |  |  |  |
| --- | --- | --- | --- |
| VARIABLES | (1) | (2) | (3) |
|  |  |  |  |
| DiD interactions |  |  |  |
| (base category: Wave Covid 1) |  |  |  |
| England x Wave 9 | -0.00107 | 0.000167 | 2.43e-06 |
|  | (0.022) | (0.022) | (0.022) |
| England x Wave 10 | -0.00392 | -0.00236 | -0.00243 |
|  | (0.028) | (0.028) | (0.028) |
| **England x Wave Covid 2** | -0.0359 | -0.0360 | -0.0361 |
|  | (0.033) | (0.033) | (0.033) |
| England x Wave Covid 3 | -0.00982 | -0.0100 | -0.0100 |
|  | (0.023) | (0.023) | (0.023) |
| England x Wave Covid 4 | 0.0236 | 0.0237 | 0.0236 |
|  | (0.023) | (0.023) | (0.023) |
| Age |  | -0.00906 | -0.00905 |
|  |  | (0.008) | (0.008) |
| Living alone |  |  | -0.00310 |
|  |  |  | (0.017) |
|  |  |  |  |
| Wave Fixed Effects | Yes | Yes | Yes |
| Individual Fixed Effects | Yes | Yes | Yes |
|  |  |  |  |
| Observations | 47,946 | 47,945 | 47,945 |
| Number of individuals | 7,991 | 7,991 | 7,991 |

NOTES: Each column reports results from a different regression, where the dependent variable is the dummy GHQ-binary2. Robust standard errors clustered at primary sampling unit in parentheses. *** p<0.01, ** p<0.05, * p<0.1 Note that the difference in observations between the final balanced sample (n=9,079) and the sample used in the regressions (n=7,991) is due to some survey respondents (1,088) having zero weights by sampling design (Kaminska & Lynn, 2019)

Table A13- DiD results, robustness check: controlling for pandemic evolution. GHQ-caseness.

|  |  |  |  |  |  |
| --- | --- | --- | --- | --- | --- |
| VARIABLES | (1) | (2) | (3) | (4) | (5) |
|  |  |  |  |  |  |
| DiD interactions |  |  |  |  |  |
| (base category: Wave Covid 1) |  |  |  |  |  |
| England x Wave 9 | 0.0339 | -0.00549 | 0.0129 | -0.0953 | 0.0630 |
|  | (0.161) | (0.164) | (0.162) | (0.180) | (0.164) |
| England x Wave 10 | -0.0545 | -0.0940 | -0.0755 | -0.184 | -0.0254 |
|  | (0.196) | (0.198) | (0.197) | (0.212) | (0.200) |
| **England x Wave Covid 2** | -0.295* | -0.246 | -0.295* | -0.376** | -0.314* |
|  | (0.173) | (0.173) | (0.170) | (0.172) | (0.169) |
| England x Wave Covid 3 | -0.194 | -0.170 | -0.175 | -0.252 | -0.226 |
|  | (0.207) | (0.207) | (0.208) | (0.207) | (0.206) |
| England x Wave Covid 4 | 0.00608 | 0.00578 | 0.0245 | -0.0857 | -0.0488 |
|  | (0.216) | (0.209) | (0.213) | (0.210) | (0.210) |
| Age | -0.0339 | -0.0322 | -0.0329 | -0.0335 | -0.0340 |
|  | (0.065) | (0.065) | (0.065) | (0.065) | (0.065) |
| Living alone | -0.0811 | -0.0792 | -0.0821 | -0.0812 | -0.0812 |
|  | (0.136) | (0.135) | (0.136) | (0.136) | (0.136) |
| Daily cases by publish date (7-day average)^†^ | -0.0157 |  |  |  |  |
|  | (0.032) |  |  |  |  |
| Daily cases by specimen date (7-day average)^†^ |  | -0.0543** |  |  |  |
|  |  | (0.024) |  |  |  |
| Cumulative cases^‡^ |  |  | -0.000520 |  |  |
|  |  |  | (0.000) |  |  |
| Daily deaths (7-day average)^†^ |  |  |  | -0.453* |  |
|  |  |  |  | (0.245) |  |
| Cumulative deaths^‡^ |  |  |  |  | 0.00266 |
|  |  |  |  |  | (0.004) |
|  |  |  |  |  |  |
| Wave Fixed Effects | Yes | Yes | Yes | Yes | Yes |
| Individual Fixed Effects | Yes | Yes | Yes | Yes | Yes |
|  |  |  |  |  |  |
| Observations | 47,945 | 47,945 | 47,945 | 47,945 | 47,945 |
| Number of individuals | 7,991 | 7,991 | 7,991 | 7,991 | 7,991 |
| NOTES: Each column reports results for a different regression, where the dependent variable is GHQ-caseness scale (0-12). Robust standard errors clustered at primary sampling unit in parentheses *** p<0.01, ** p<0.05, * p<0.1. ^†^Daily cases (or deaths) were measured as the average of new cases (or deaths) of the last 7 days prior to the day of responding the survey, at each survey wave and region. ^‡^Cumulative cases (or deaths) measures the total number of cases (or deaths) up to the day before responding the survey, at each survey wave and region. More details about these variables and how they were inserted in the model can be found in Appendix D. Note that the difference in observations between the final balanced sample (n=9,079) and the sample used in the regressions (n=7,991) is due to some survey respondents (1,088) having zero weights by sampling design (Kaminska & Lynn, 2019). | | | | | |

Table A14- DiD results, robustness check: controlling for pandemic evolution. GHQ-likert.

|  |  |  |  |  |  |
| --- | --- | --- | --- | --- | --- |
| VARIABLES | (1) | (2) | (3) | (4) | (5) |
|  |  |  |  |  |  |
| DiD interactions |  |  |  |  |  |
| (base category: Wave Covid 1) |  |  |  |  |  |
| England x Wave 9 | 0.462 | 0.459 | 0.490 | 0.398 | 0.620* |
|  | (0.319) | (0.331) | (0.327) | (0.351) | (0.336) |
| England x Wave 10 | -0.0776 | -0.0817 | -0.0504 | -0.142 | 0.0796 |
|  | (0.342) | (0.348) | (0.344) | (0.365) | (0.349) |
| **England x Wave Covid 2** | -0.462* | -0.380 | -0.464* | -0.490* | -0.451* |
|  | (0.256) | (0.257) | (0.253) | (0.269) | (0.258) |
| England x Wave Covid 3 | -0.0314 | 0.0297 | -0.0589 | -0.0397 | -0.0572 |
|  | (0.308) | (0.309) | (0.303) | (0.314) | (0.309) |
| England x Wave Covid 4 | 0.318 | 0.384 | 0.289 | 0.311 | 0.278 |
|  | (0.289) | (0.286) | (0.281) | (0.298) | (0.289) |
| Age | -0.0748 | -0.0726 | -0.0762 | -0.0739 | -0.0739 |
|  | (0.123) | (0.123) | (0.122) | (0.123) | (0.123) |
| Living alone | -0.217 | -0.214 | -0.215 | -0.216 | -0.216 |
|  | (0.227) | (0.227) | (0.226) | (0.227) | (0.227) |
| Daily cases by publish date (7-day average)^†^ | 0.0199 |  |  |  |  |
|  | (0.052) |  |  |  |  |
| Daily cases by specimen date (7-day average)^†^ |  | -0.0522 |  |  |  |
|  |  | (0.041) |  |  |  |
| Cumulative cases^‡^ |  |  | 0.000719 |  |  |
|  |  |  | (0.001) |  |  |
| Daily deaths (7-day average)^†^ |  |  |  | -0.331 |  |
|  |  |  |  | (0.370) |  |
| Cumulative deaths^‡^ |  |  |  |  | 0.00825 |
|  |  |  |  |  | (0.006) |
|  |  |  |  |  |  |
| Wave Fixed Effects | Yes | Yes | Yes | Yes | Yes |
| Individual Fixed Effects | Yes | Yes | Yes | Yes | Yes |
|  |  |  |  |  |  |
| Observations | 47,945 | 47,945 | 47,945 | 47,945 | 47,945 |
| Number of individuals | 7,991 | 7,991 | 7,991 | 7,991 | 7,991 |
| NOTES: Each column reports results for a different regression, where the dependent variable is GHQ-likert scale (0-36). Robust standard errors clustered at primary sampling unit in parentheses *** p<0.01, ** p<0.05, * p<0.1. ^†^Daily cases (or deaths) were measured as the average of new cases (or deaths) of the last 7 days prior to the day of responding the survey, at each survey wave and region. ^‡^Cumulative cases (or deaths) measures the total number of cases (or deaths) up to the day before responding the survey, at each survey wave and region. More details about these variables and how they were inserted in the model can be found in Appendix D. Note that the difference in observations between the final balanced sample (n=9,079) and the sample used in the regressions (n=7,991) is due to some survey respondents (1,088) having zero weights by sampling design (Kaminska & Lynn, 2019). | | | | | |

Table A15- DiD results, robustness check: controlling for pandemic evolution. GHQ-binary.

|  |  |  |  |  |  |
| --- | --- | --- | --- | --- | --- |
| VARIABLES | (1) | (2) | (3) | (4) | (5) |
|  |  |  |  |  |  |
| DiD interactions |  |  |  |  |  |
| (base category: Wave Covid 1) |  |  |  |  |  |
| England x Wave 9 | 0.00849 | 0.0151 | 0.0157 | 0.0175 | 0.0304 |
|  | (0.027) | (0.026) | (0.026) | (0.027) | (0.026) |
| England x Wave 10 | -0.0494 | -0.0428 | -0.0421 | -0.0404 | -0.0275 |
|  | (0.060) | (0.059) | (0.059) | (0.060) | (0.060) |
| **England x Wave Covid 2** | -0.0730*** | -0.0657*** | -0.0674*** | -0.0648*** | -0.0671*** |
|  | (0.023) | (0.022) | (0.021) | (0.021) | (0.021) |
| England x Wave Covid 3 | -0.0281 | -0.0203 | -0.0236 | -0.0197 | -0.0257 |
|  | (0.031) | (0.031) | (0.031) | (0.031) | (0.031) |
| England x Wave Covid 4 | -0.0220 | -0.00867 | -0.0130 | -0.00763 | -0.0175 |
|  | (0.030) | (0.027) | (0.027) | (0.026) | (0.026) |
| Age | -0.00376 | -0.00362 | -0.00374 | -0.00365 | -0.00357 |
|  | (0.009) | (0.009) | (0.009) | (0.009) | (0.009) |
| Living alone | 0.0191 | 0.0191 | 0.0192 | 0.0191 | 0.0191 |
|  | (0.022) | (0.022) | (0.022) | (0.022) | (0.022) |
| Daily cases by publish date (7-day average)^†^ | 0.00695 |  |  |  |  |
|  | (0.006) |  |  |  |  |
| Daily cases by specimen date (7-day average)^†^ |  | -0.000341 |  |  |  |
|  |  | (0.004) |  |  |  |
| Cumulative cases^‡^ |  |  | 4.46e-05 |  |  |
|  |  |  | (0.000) |  |  |
| Daily deaths (7-day average)^†^ |  |  |  | 0.00892 |  |
|  |  |  |  | (0.034) |  |
| Cumulative deaths^‡^ |  |  |  |  | 0.000906** |
|  |  |  |  |  | (0.000) |
|  |  |  |  |  |  |
| Wave Fixed Effects | Yes | Yes | Yes | Yes | Yes |
| Individual Fixed Effects | Yes | Yes | Yes | Yes | Yes |
|  |  |  |  |  |  |
| Observations | 47,945 | 47,945 | 47,945 | 47,945 | 47,945 |
| Number of individuals | 7,991 | 7,991 | 7,991 | 7,991 | 7,991 |
| NOTES: Each column reports results for a different regression, where the dependent variable is the dummy variable GHQ-binary. Robust standard errors clustered at primary sampling unit in parentheses *** p<0.01, ** p<0.05, * p<0.1. ^†^Daily cases (or deaths) were measured as the average of new cases (or deaths) of the last 7 days prior to the day of responding the survey, at each survey wave and region. ^‡^Cumulative cases (or deaths) measures the total number of cases (or deaths) up to the day before responding the survey, at each survey wave and region. More details about these variables and how they were inserted in the model can be found in Appendix D. Note that the difference in observations between the final balanced sample (n=9,079) and the sample used in the regressions (n=7,991) is due to some survey respondents (1,088) having zero weights by sampling design (Kaminska & Lynn, 2019). | | | | | |

Table A16- DiD results, robustness check: controlling for pandemic evolution. GHQ-binary2.

|  |  |  |  |  |  |
| --- | --- | --- | --- | --- | --- |
| VARIABLES | (1) | (2) | (3) | (4) | (5) |
|  |  |  |  |  |  |
| DiD interactions |  |  |  |  |  |
| (base category: Wave Covid 1) |  |  |  |  |  |
| England x Wave 9 | -0.00129 | -0.00219 | -0.000513 | -0.00597 | 0.00563 |
|  | (0.024) | (0.022) | (0.022) | (0.024) | (0.023) |
| England x Wave 10 | -0.00371 | -0.00464 | -0.00295 | -0.00841 | 0.00320 |
|  | (0.029) | (0.028) | (0.028) | (0.029) | (0.029) |
| **England x Wave Covid 2** | -0.0374 | -0.0302 | -0.0346 | -0.0395 | -0.0365 |
|  | (0.034) | (0.034) | (0.033) | (0.033) | (0.033) |
| England x Wave Covid 3 | -0.0115 | -0.00631 | -0.00684 | -0.0122 | -0.0120 |
|  | (0.024) | (0.023) | (0.023) | (0.023) | (0.023) |
| England x Wave Covid 4 | 0.0211 | 0.0263 | 0.0280 | 0.0204 | 0.0204 |
|  | (0.026) | (0.023) | (0.023) | (0.023) | (0.023) |
| Age | -0.00907 | -0.00887 | -0.00894 | -0.00901 | -0.00903 |
|  | (0.008) | (0.008) | (0.008) | (0.008) | (0.008) |
| Living alone | -0.00311 | -0.00292 | -0.00318 | -0.00310 | -0.00310 |
|  | (0.017) | (0.017) | (0.017) | (0.017) | (0.017) |
| Daily cases by publish date (7-day average)^†^ | 0.00133 |  |  |  |  |
|  | (0.006) |  |  |  |  |
| Daily cases by specimen date (7-day average)^†^ |  | -0.00494 |  |  |  |
|  |  | (0.004) |  |  |  |
| Cumulative cases^‡^ |  |  | -4.70e-05 |  |  |
|  |  |  | (0.000) |  |  |
| Daily deaths (7-day average)^†^ |  |  |  | -0.0238 |  |
|  |  |  |  | (0.033) |  |
| Cumulative deaths^‡^ |  |  |  |  | 0.000337 |
|  |  |  |  |  | (0.000) |
|  |  |  |  |  |  |
| Wave Fixed Effects | Yes | Yes | Yes | Yes | Yes |
| Individual Fixed Effects | Yes | Yes | Yes | Yes | Yes |
|  |  |  |  |  |  |
| Observations | 47,945 | 47,945 | 47,945 | 47,945 | 47,945 |
| Number of individuals | 7,991 | 7,991 | 7,991 | 7,991 | 7,991 |
| NOTES: Each column reports results for a different regression, where the dependent variable is the dummy variable GHQ-binary. Robust standard errors clustered at primary sampling unit in parentheses *** p<0.01, ** p<0.05, * p<0.1. ^†^Daily cases (or deaths) were measured as the average of new cases (or deaths) of the last 7 days prior to the day of responding the survey, at each survey wave and region. ^‡^Cumulative cases (or deaths) measures the total number of cases (or deaths) up to the day before responding the survey, at each survey wave and region. More details about these variables and how they were inserted in the model can be found in Appendix D. Note that the difference in observations between the final balanced sample (n=9,079) and the sample used in the regressions (n=7,991) is due to some survey respondents (1,088) having zero weights by sampling design (Kaminska & Lynn, 2019). | | | | | |

Table A17- DiD results, robustness check 2: Timing of Scotland easing restrictions

|  | (1) | (2) | (3) | (4) |
| --- | --- | --- | --- | --- |
| VARIABLES | GHQ-caseness | GHQ-likert | GHQ-binary | GHQ-binary2 |
|  |  |  |  |  |
| DiD interactions |  |  |  |  |
| (base category: Wave Covid 1) |  |  |  |  |
| England x Wave 9 | 0.126 | 0.692* | 0.006 | 0.009 |
|  | (0.207) | (0.415) | (0.031) | (0.029) |
| England x Wave 10 | -0.172 | -0.238 | -0.084 | -0.023 |
|  | (0.252) | (0.438) | (0.080) | (0.033) |
| **England x Wave Covid 2** | -0.396* | -0.513 | -0.076*** | -0.051 |
|  | (0.209) | (0.317) | (0.027) | (0.043) |
| England x Wave Covid 3 | -0.143 | 0.171 | -0.028 | -0.009 |
|  | (0.267) | (0.354) | (0.039) | (0.028) |
| England x Wave Covid 4 | 0.059 | 0.575* | -0.021 | 0.022 |
|  | (0.269) | (0.340) | (0.031) | (0.027) |
| Age | -0.023 | -0.053 | -0.002 | -0.009 |
|  | (0.066) | (0.125) | (0.009) | (0.008) |
| Living alone | -0.076 | -0.220 | 0.019 | -0.002 |
|  | (0.137) | (0.229) | (0.022) | (0.017) |
|  |  |  |  |  |
| Wave Fixed Effects | Yes | Yes | Yes | Yes |
| Individual Fixed Effects | Yes | Yes | Yes | Yes |
|  |  |  |  |  |
| Observations | 46,451 | 46,451 | 46,451 | 46,451 |
| Number of individuals | 7,742 | 7,742 | 7,742 | 7,742 |
| NOTES: Each column reports results for a different regression. In Column (1) the dependent variable is the GHQ-caseness scale (0-12), in Column (2) the GHQ-likert scale (0-36), in Column (3) the dummy variable GHQ-binary and in Column(4) the dummy variable GHQ-binary2. Robust standard errors clustered at primary sampling unit in parentheses *** p<0.01, ** p<0.05, * p<0.1. For Scotland, we only used the subsample of individuals who,at Covid survey wave 2, responded before the day 29/05 where some restrictions in Scotland were lifted. They account for 68% of our final sample in Scotland (622 out of 915 individuals) | | | | |

**Appendix B. Flowchart of sample selection**

Baseline sample Wave 9

(n=36,056)

Unobserved at Wave 10

(n=5,113). Main reasons: refusal (30%), non-contacted (25.3%), death (4.5%), moved from household (4%) illness (1.9%), moved out of the country (1.5%), other reasons (33%)

Observed Waves 9-10

(n=30,943)

Unobserved at Wave CS1

(n=15,311). Main reasons: non-response (95.3%), death (0.2%), other reasons (4.5%)

Observed Waves 9-CS1

(n=15,632)

Unobserved at Wave CS2

(n=3,107). Main reasons: non-response (98%), death (0.1%), other reasons (1.9%)

Observed Waves 9-CS2

(n=12,525)

Unobserved at Wave CS3

(n=1,024). Main reasons: non-response (99.2%), death (0.1%), other reasons (0.7%)

Observed Waves 9-CS3

(n=11,501)

Unobserved at Wave CS4

(n=834). Main reasons: non-response (99.8%), other reasons (0.7%)

Balanced sample Waves 9-CS4

(n=10,667)

Non-response in General Health Questionnaire (GHQ)

(n=564)

Balanced sample Waves 9-CS4 responded to General Health Questionnaire (GHQ)

(n=10,103)

Living in Wales or Northern Ireland during Covid Survey Wave 2 (May 2020)

(n=1,024)

Balanced sample Waves 9-CS4 responded to General Health Questionnaire (GHQ) & Living in England or Scotland during Wave 2 Covid (May 2020)

(n=9,079)

* “Wave CS”= Covid Survey Wave

**Appendix C. Inverse probability weights**

In order to construct the weights of our model we first, we first use a response model by estimating the probability of responding in all waves of the GHQ questionnaire (i.e., taking part of the balanced sample) as a function of observable variables at the baseline wave (i.e., wave 9). In UKHLS the GHQ questionnaire was only completed by those responding the self-completion adult interview. Information from other individuals taking part of UKHLS was actually provided by a proxy respondent. The latter group did not complete the GHQ questionnaire (Institute for Social and Economic Research, 2020). Hence, our final balanced sample is formed by those completing the self-completion adult interview, and therefore present information on GHQ in all waves. Then Inverse Probability Weights (IPW) are formed as the inverse of the predicted probability of responding in the balanced sample. Lastly, we multiply these weights by the cross-sectional weights from the wave 9 provided by UKHLS. Below, we elaborate the construction of these weights, that were created following the instructions provided by the UK household Longitudinal Survey (Kaminska & Lynn, 2019).

Our response model is based on the following probit model:

$$r_{i}=X_{i}\beta+\varepsilon_{i}$$

Individuals included in the response model are all wave 9 respondents who have non-zero weights in the cross-sectional weights of wave 9, and did not die or move out of the country during our period of analysis.

$r_{ic}$ equals 1 if individual $i$ responded in al waves to the GHQ questionnaire (i.e., takes part of the balanced sample), and zero otherwise. $X_{i}$ is a vector formed by the following variables measured at wave 9: age (continuous) , age squared (continuous), female (dummy), education level (categorical: *less than O-level*, *O-level, higher than A-level, not available*), poor self-reported health (dummy), access to internet (categorical: *yes*, *no*, *not* *available*), smoker (dummy), labour market status (*employed, unemployed, student, inactive, homemaker*, *not available*), nation (categorical: *England, Wales, Scotland, Northern Ireland, not available*), household quintil (categorical: *quintil 1, quintil 2, quintil 3, quintil 4, quintil 5, not available*). Results of the response model are reported in Table B1. Important to note that the probability of response (and therefore attrition) was not different across England and Scotland after controlling for the rest of the predictors, since the coefficient of the Scotland dummy is not significant.

IPWs are then formed by the inverse of the predicted probability of being part of the the balanced sample ($\hat{r}_{i}$).

$${IPW}_{i}= \frac{1}{\hat{r}_{i}}$$

Lastly, to get our final weights, we multiply the IPWs by the cross-sectional weights of wave 9 provided by UKHLS (Kaminska & Lynn, 2019). Concretely, the cross-sectional weights of wave 9 that best suit for our analysis are named “i_indscui_xw”, and make the sample representative of individuals who responded the self-completion adult interview at wave 9.

$${{Final weights}_{i}= {IPW}_{i} x i\_indscui\_xw}_{i}$$

Table C1- Response model (probit model) results.

| VARIABLES | Prob (response in all waves) |
| --- | --- |
| Age | 0.0885*** |
|  | (0.003) |
| Age square | -0.000793*** |
|  | (0.000) |
| Female | 0.136*** |
|  | (0.017) |
| Education level (Base category: Less than O-level or equivalent) |  |
| O -level, A - level or equivalent | 0.202*** |
|  | (0.025) |
| Higher than A - level | 0.292*** |
|  | (0.024) |
| Not available | 0.0601** |
|  | (0.029) |
| Self-reported health (Base category: Good or better) |  |
| Fair or Poor | -0.210*** |
|  | (0.022) |
| smoker | -0.324*** |
|  | (0.027) |
| Access to internet (Base category: Yes) |  |
| No access | -1.093*** |
|  | (0.055) |
| Not available | -0.421* |
|  | (0.221) |
| Labour market status (Base category: employed) |  |
| unemployed | -0.0799 |
|  | (0.052) |
| student | 0.168*** |
|  | (0.050) |
| inactive (retired or disabled) | 0.277*** |
|  | (0.028) |
| homemaker | -0.151*** |
|  | (0.046) |
| Not available | -0.146 |
|  | (0.404) |
| Nation of residence (Base category: England) |  |
| Wales | -0.0862** |
|  | (0.034) |
| Scotland | 0.0128 |
|  | (0.030) |
| Northern Ireland | -0.350*** |
|  | (0.038) |
| Not available | 0.232 |
|  | (0.482) |
| Income quintil (Base category: Quintil 1) |  |
| Quintil 2 | 0.107*** |
|  | (0.029) |
| Quintil 3 | 0.235*** |
|  | (0.028) |
| Quintil 4 | 0.258*** |
|  | (0.028) |
| Quintil 5 | 0.339*** |
|  | (0.029) |
| Not available | 0.345 |
|  | (0.223) |
| NOTES: Standard errors in parentheses. *** p<0.01, ** p<0.05, * p<0.1. Number of observations: 27,850 | |

**Appendix D. Data on COVID-19 cases and deaths.**

The data for COVID-19 cases and deaths used in the robustness checks (Tables A12-A14 of Appendix A) come from UK government official statistics (<https://coronavirus.data.gov.uk/details/download>). The availability of the data slightly varies for the English regions and for Scotland. In Table D1 below, we summarize the availability by region. Based on this, we used the following measures to control for the impact of the pandemic in the robustness check, always trying to maximize the comparability of the data from England and Scotland:

- Daily cases by publish date (7-day rolling average)

- Daily cases by specimen date (7-day rolling average)

- Cumulative cases. Based on the specimen date for both English regions and Scotland.

- Daily deaths (7-day rolling average). Based on date of death for English regions, and on publish date for Scotland.

- Cumulative deaths. Based on date of death for English regions, and on publish date for Scotland.

These variables are measured at regional level, which is the lowest level at which information was available for both UKHLS and COVID-19 data. There were 10 regions: Scotland and 9 regions of England (South East, London, North West, East of England, West Midlands, South West, Yorkshire and the Humber, East Midlands and North East).

Values of these variables are inserted in the model for each individual as of the day before of the responding date, based on respondent’s region of residence by April 2020 and date of responding. We therefore assume that the respondent is aware of the epidemic evolution as of the day before of responding the survey.

For daily cases and daily deaths, we use the 7-day rolling average in order to avoid fluctuations in the reporting of cases and deaths at the different days of the week when participants responded the survey.

Table D1- Summary Table of COVID-19 data availability and use in the robustness check model

|  | England regions | Scotland |
| --- | --- | --- |
| Daily cases (7-day rolling average) |  |  |
| by publish date | x | x |
| by specimen date | x | x |
| Cumulative cases |  |  |
| by publish date |  | x |
| by specimen date | x | x |
| Daily deaths (7-day rolling average) |  |  |
| by publish date |  | x |
| by death date | x |  |
| Cumulative deaths |  |  |
| by publish date |  | x |
| by death date | x |  |

NOTES: Data available for the period under study is marked with an “x”. Data finally included in the models of the robustness check (Tables A12-A14) are highlighted in gray.

**Appendix E. Triple difference model**

We allowed the DiD coefficient to vary across groups including a triple interaction. (England x Waves x socioeconomic group). In order for the triple interaction to be meaningful, we also included the double interaction (Waves x socio-economic group). Therefore, our model effectively turns into a triple difference model as follows:

${{GHQ}_{i,t} = \beta}_{0}+ \beta_{1}{Wave}_{t} +\beta_{2}\left( {England}_{i} x {Wave}_{t} \right)+\beta_{3}\left( {SE group}_{i} x {Wave}_{t} \right) +\beta_{4}\left( {SE group}_{i} x England x {Wave}_{t} \right)+ X_{it}\gamma+ \alpha_{i}+\mu_{i,t}$

${SE group}_{i}$ is a dummy variable for the respective socio-economic group: gender (equals 1 if female, 0 if male), education (equals 1 if A-level or lower, 0 if higher than A – level), household income (equals 1 if below median, 0 if above median), household earning loss by April 2020 (equals 1 if Yes, 0 if No), self-reported financial situation by April 2020 (equals 1 if bad, 0 if good). Since we have 3 age groups, ${SE group}_{i}$ is a categorical variable with age 16-34, age 35-64, and age 65+ (reference category). The other variables are the same as those in Equation 1.We then run this triple difference model for each SE division: i.e.: one for gender, other for education, etc.

The triple interaction $\beta_{4}$ at Covid survey wave 2 (late May 2020) measures whether the effect of easing restrictions is significantly different across socio-economic groups.

The double interaction $\beta_{2}\left( {England}_{i} x {Wave \left( Covid survey wave 2 \right)}_{t} \right)$ measures the effect of easing restrictions in the reference socioeconomic group (i.e.: when the SE group dummy is equal zero), whereas the effect on the other SE group (i.e.: when SE group dummy equals one) is measured by the linear combination of $\beta_{2}\left( {England}_{i} x {Wave \left( Covid survey wave 2 \right)}_{t} \right)$ + $\beta_{4}\left( {SE group}_{i} x England x {Wave \left( Covid survey wave \right)}_{t} \right)$

Full results of the triple difference model are reported in Tables E1 and E2 below.

**Table E1 – Results from the triple difference model.**

|  | (1) | (2) | (3) | (4) | (5) |
| --- | --- | --- | --- | --- | --- |
| VARIABLES | Gender | Education | Income | Earning loss | Financial situation |
| SE group x Wave 9 | -0.587* | 0.460 | -0.199 | -0.0373 | 0.0207 |
|  | (0.309) | (0.347) | (0.369) | (0.368) | (0.364) |
| SE group x Wave 10 | -0.944** | 0.428 | -0.0485 | 0.00993 | -0.191 |
|  | (0.382) | (0.390) | (0.417) | (0.384) | (0.475) |
| SE group x Wave Covid 2 | -0.679** | 0.763** | 0.156 | 0.277 | 0.635 |
|  | (0.294) | (0.340) | (0.329) | (0.404) | (0.397) |
| SE group x Wave Covid 3 | -0.278 | 0.113 | -0.0945 | -0.217 | 0.233 |
|  | (0.425) | (0.391) | (0.343) | (0.422) | (0.527) |
| SE group x Wave Covid 4 | -0.757** | 0.463 | 0.169 | -0.235 | 0.266 |
|  | (0.376) | (0.324) | (0.358) | (0.379) | (0.514) |
| DiD interactions |  |  |  |  |  |
| (base category: Wave Covid 1) |  |  |  |  |  |
| England x Wave 9 | 0.164 | 0.0667 | -0.381 | 0.0910 | 0.0461 |
|  | (0.210) | (0.326) | (0.277) | (0.218) | (0.204) |
| England x Wave 10 | -0.203 | -0.0408 | -0.156 | 0.167 | -0.0217 |
|  | (0.295) | (0.298) | (0.306) | (0.257) | (0.200) |
| **England x Wave Covid 2** | -0.408 | 0.153 | -0.225 | -0.0895 | -0.0465 |
|  | (0.254) | (0.197) | (0.229) | (0.167) | (0.141) |
| England x Wave Covid 3 | -0.106 | -0.0209 | -0.306 | -0.203 | -0.0526 |
|  | (0.330) | (0.240) | (0.211) | (0.235) | (0.190) |
| England x Wave Covid 4 | -0.0599 | 0.505** | -0.0168 | -0.00735 | 0.148 |
|  | (0.307) | (0.254) | (0.239) | (0.260) | (0.171) |
| Triple difference interactions |  |  |  |  |  |
| SE group x England x Wave 9 | -0.234 | -0.108 | 0.594 | -0.223 | -0.106 |
|  | (0.344) | (0.391) | (0.397) | (0.403) | (0.416) |
| SE group x England x Wave 10 | 0.297 | -0.135 | 0.224 | -0.719* | -0.257 |
|  | (0.409) | (0.426) | (0.445) | (0.417) | (0.516) |
| **SE group x England x Wave Covid 2** | 0.216 | -0.889** | -0.0940 | -0.670 | -0.871** |
|  | (0.316) | (0.358) | (0.343) | (0.423) | (0.434) |
| SE group x England x Wave Covid 3 | -0.176 | -0.188 | 0.272 | -0.0294 | -0.570 |
|  | (0.441) | (0.413) | (0.362) | (0.443) | (0.552) |
| SE group x England x Wave Covid 4 | 0.109 | -0.544 | 0.0201 | -0.0524 | -0.611 |
|  | (0.395) | (0.349) | (0.375) | (0.405) | (0.541) |
| Age | -0.0337 | -0.0334 | -0.0228 | -0.0321 | -0.0379 |
|  | (0.065) | (0.069) | (0.064) | (0.065) | (0.065) |
| Living alone | -0.0861 | -0.0565 | -0.0850 | -0.0875 | -0.0783 |
|  | (0.137) | (0.139) | (0.138) | (0.136) | (0.135) |
|  |  |  |  |  |  |
| Wave Fixed Effects | Yes | Yes | Yes | Yes | Yes |
| Individual Fixed Effects | Yes | Yes | Yes | Yes | Yes |
| Observations | 47,945 | 42,821 | 47,250 | 47,897 | 47,927 |
| Number of individuals | 7,991 | 7,137 | 7,875 | 7,983 | 7,988 |

NOTES: This table reports results from the triple difference model with GHQ-caseness as dependent variable. Each column reports results from a different regression, using the the dummy variable of the corresponding socioeconomic group (SE group) in the triple interaction. In column (1) *SEgroup* equals one for female, zero for males. In column (2) *SEgroup* equals one for A-level or lower education, zero for higher than A-level. In column (3) *SEgroup* equals one for a household income below the median in wave 10, zero for above the median. In column (4) *SEgroup* equals one for having suffered an earning loss in April 2020 with respect to March 2020, zero otherwise. In column (4) *SEgroup* equals one for those who reported a bad self-reported financial situation by April 2020 and zero for those who reported a good financial situation. The latter category is based on the question “How well would you say you yourself are managing financially these days?”; those who responded “Just about getting by”, “Finding it quite difficult” or “Finding it very difficult” were classified as Bad financial situation, whereas those who responded “living comfortably” or “doing alright” were classified as Good financial situation. Robust standard errors clustered at primary sampling unit in parentheses *** p<0.01, ** p<0.05, * p<0.1.

**Table E2– Results from the triple difference model (II): age groups**

|  | (1) |
| --- | --- |
| VARIABLES | Age groups |
|  |  |
| Age 16-30 x Wave 9 | -1.071** |
|  | (0.470) |
| Age 35-64 x Wave 9 | 0.0989 |
|  | (0.259) |
| Age 16-30 x Wave 10 | -0.990 |
|  | (0.619) |
| Age 35-64 x Wave 10 | -0.438 |
|  | (0.362) |
| Age 16-30 x Wave Covid 2 | 0.224 |
|  | (0.399) |
| Age 35-64 x Wave Covid 2 | 0.198 |
|  | (0.263) |
| Age 16-30 x Wave Covid 3 | 0.0297 |
|  | (0.638) |
| Age 35-64 x Wave Covid 3 | -0.473 |
|  | (0.437) |
| Age 16-30 x Wave Covid 4 | -1.143* |
|  | (0.594) |
| Age 35-64 x Wave Covid 4 | -0.937** |
|  | (0.431) |
| DiD interactions |  |
| (base category: Wave Covid 1) |  |
| England x Wave 9 | -0.0789 |
|  | (0.217) |
| England x Wave 10 | -0.427 |
|  | (0.321) |
| **England x Wave Covid 2** | -0.0112 |
|  | (0.099) |
| England x Wave Covid 3 | -0.251 |
|  | (0.373) |
| England x Wave Covid 4 | -0.536 |
|  | (0.388) |
|  |  |
| Table continues next page …. |  |
|  |  |
| Triple difference interactions  (base category: Wave Covid 1) |  |
| Age 16-30 x England x Wave 9 | 0.712 |
|  | (0.519) |
| Age 35-64 x England x Wave 9 | -0.0396 |
|  | (0.289) |
| Age 16-30 x England x Wave 10 | 0.667 |
|  | (0.655) |
| Age 35-64 x England x Wave 10 | 0.464 |
|  | (0.388) |
| **Age 16-30 x England x Wave Covid 2** | -0.596 |
|  | (0.433) |
| **Age 35-64 x England x Wave Covid 2** | -0.292 |
|  | (0.278) |
| Age 16-30 x England x Wave Covid 3 | -0.541 |
|  | (0.665) |
| Age 35-64 x England x Wave Covid 3 | 0.352 |
|  | (0.449) |
| Age 16-30 x England x Wave Covid 4 | 0.596 |
|  | (0.622) |
| Age 35-64 x England x Wave Covid 4 | 0.809* |
|  | (0.442) |
|  |  |
| Age | -0.0284 |
|  | (0.064) |
| Living alone | -0.0850 |
|  | (0.137) |
|  |  |
| Observations | 47,945 |
| Number of pidp | 7,991 |
| R-squared | 0.034 |

NOTES: This table reports results from the triple difference model with GHQ-caseness as dependent variable. The base category for the age group categorical variable if 65+. Robust standard errors clustered at primary sampling unit in parentheses *** p<0.01, ** p<0.05, * p<0.1.
